# Acknowledgment to Reviewers of *Biomolecules* in 2020

**DOI:** 10.3390/biom11020151

**Published:** 2021-01-25

**Authors:** Biomolecules Editorial Office

**Affiliations:** MDPI AG, St. Alban-Anlage 66, 4052 Basel, Switzerland

Peer review is the driving force of journal development, and reviewers are gatekeepers who ensure that *Biomolecules* maintains its standards for the high quality of its published papers. Thanks to the cooperation of our reviewers, in 2020, the median time to first decision was 17 days and the median time to publication was 39 days. The editors would like to express their sincere gratitude to the following reviewers for their precious time and dedication, regardless of whether the papers were finally published:
Abate, GiuliaLisi, LuciaAbbott, Jessica K.Lisuzzo, LorenzoAbdelmohsen, Usama RamadanLiszkowska, JoannaAbenavoli, LudovicoLiu, Bing-LanAbeykoon, JithmaLiu, ChaoxingAblashi, DharamLiu, ChunmingAbomoelak, BassamLiu, FuguoAbraham, Wolf-RainerLiu, GangAbramson, Hanley N.Liu, GuangleiAbushouk, AbdelrahmanLiu, Hai-yangAccardo, AngeloLiu, HongxiangAcquas, ElioLiu, JunliAdamczyk, BartoszLiu, LingAdamczyk-Grochala, JagodaLiu, MingxianAdamek, JakubLiu, TaoAdamek, MikolajLiu, WeiAdams, JamesLiu, XiangleiAdams, StephenLiu, YonggangAdamska, AgnieszkaLiu, Yung-ChuanAdar, SheeraLiu, ZhipingÅdén, JörgenLiWang, PatriciaAdhikari, NeetaLiwo, AdamAdriana, Estrada-BernalLlama-Palacios, AranchaAffolter, MarkusLlanes, AlejandroAffourtit, CharlesLlavero, FranciscoAgamennone, MariangelaLocascio, AnnamariaAggeli, Ioanna-KaterinaLocatelli, MarcelloAghaeepour, NimaLock, AntoniaAgostino, MarkLoeffler, Christopher R.Agüero-Chapin, GuillerminLogan, DerekAhmad, FahimLoghin, FeliciaAhn, JuheeLoh, StewartAhn, Yeong HeeLoid, JosefAi, YongLoinard, CélineAiello, FrancescaLombardi, TommasoAimanianda, VishukumarLombardi, Vincent C.Akai, HiroyukiLonardo, AmedeoAkbar, MohammedLong, YuchenAkimov, Sergey A.Longley, DanAkopova, TatianaLonni, Audrey A. Stinghen GarciaAlam, ShafiulLopes, Coeli M.B.Alarcón-Riquelme, Marta E.Lopes, Ricardo JorgeAlbani, MariaLópez Fernández, HugoAlbarella, SaraLópez Martínez, JuanAlbericio, FernandoLopez, César A.Albers, Sonja VerenaLópez, Kelly Johana FigueroaAlduina, RosaLopez, VictorAlekseeva, AnnaLópez-Camarillo, CésarAleksova, AnetaLopez-Canul, MarthaAlessenko, Alice V.López-Cebral, RitaAlexandri, MariaLópez-Hernández, Francisco J.Alfano, DanielaLopez-Martin, ManuelAl-Hilaly, YoussraLopez-Soto, AlejandroAli, Hayssam M.Loring, Ralph H.Alini, MauroLosurdo, GiuseppeAlipieva, KalinaLothier, JérémyAllen, ClaireLouis, CorentinAllers, ThorstenLoureiro, Joana A.Allmang, ChristineLourenço, AnaAlloatti, GiuseppeLöw, PéterAlmajano, María PilarLoza-Mejía, Marco A.Almeida-Paes, RodrigoLu, Jeng-WeiAlonso, Alejandra D.Lu, Wan-JungAlonso-Alconada, DanielLubin-Germain, NadègeAlseekh, SalehLucafò, MariannaAltamirano-Bustamante, Myriam M.Lucas, Andrew T.Altegoer, FlorianLuce-Fedrow, AlisonAltieri, FabioLucentini, LiviaAltintas, Mehmet MLuchinat, EnricoAltman, AmnonLudidi, NdikoAltman, Ryan A.Ludwikõw, AgnieszkaAlvarez Fernandez, María AntoniaLugao, AdemarAlves E Silva, Thiago LuizLui, Edmund Man KingAlves, Ana CatarinaLuigi, PalmieriAlzubaidi, LaithLuisa, BenussiAmaral, JoanaŁukasz Konefał, RafałAmaro, HelenaLund, PeteAmato, Dominick J.Lundström, ClaesAmato, FeliceLuongo, LivioAmbite, InesLupattelli, PaoloAmbrosino, ConcettaLutfalla, GeorgesAmbrosio, SantiagoLuthra, PriyaAmeln, Anne Klotzsche-vonLuwor, RodneyAmes, James BLux, ThomasAmigo, LourdesLux-Battistelli, ChristineAmir, RachelLv, HuixiongAmodio, NicolaŁyczko, JacekAmorós, AsunciónLye, GaryAmrani, YassineLymperopoulos, AnastasiosAn, HongLynch, MarinaAn, Jeung HeeLyons, ShawnAnastasiadi, MariaLyukmanova, Ekaterina N.Anberitha Matthews, AnberithaM. Aizpurua, JesúsAnchordoquy, TomMa, ChengbangAncuceanu, RobertMa, GuojiaAnděra, LadislavMa, Jiyan Anders, JuanitaMa, WeiAndersen, Tonni GrubeMacauley, Matthew S.Anderson, Marilyn A.Maccallini, CristinaAndersson, EmmaMacchi, ChiaraAndré, VâniaMacDiarmid, Colin W.Andreas, Loren BMacek Jílková, ZuzanaAndreev, Yaroslav A.Macnaughten, MeghanAndreoni, FrancescaMaddelein, Marie-LiseAndres, Maria FeMadine, JillAndrés-León, EduardoMadine, JillianAndrisano, VincenzaMadrid, AlejandroAndrius, KazlauskasMadureira, PatriciaAngeletti, MauroMaechler, PierreAngioni, AlbertoMaeng, Sung HoAngius, FabrizioMaestro, BeatrizAnne, JozefMaffei, AngeloAnni, VedelerMaftah, AbderrahmanAnnibal, AndreaMafu, SibongileAnsari, D. Mohd. AzamMagarlamov, Timur Yu.Antalíková, JanaMaggi, DavideAntinozzi, CristinaMagistrato, AlessandraAntipova, VeronicaMagne, DavidAntoce, Arina OanaMagnus, MarcinAntognelli, CinziaMagyar, CsabaAntoni, GunnarMahavadi, SunilaAntoniali, GiuliaMahmassani, Ziad S.Antoniassi, RosemarMahmoud, AymanAntoniewska, AgataMains, Richard E.Antony, AntoniouMaisch, BernhardAntonyuk, SvetlanaMaity, ShuvadeepAppendino, GiovanniMajtan, TomasAprodu, IulianaMajumdar, SusrutaAprotosoaie, Ana ClaraMakarevich, PavelAquilano, KatiaMakarova, Kira SArai, ToshiroMakhutova, OlesiaArakawa, KenjiMakoto, HirayamaAraújo, Maria Eduarda MachadoMakowska, KrystynaArbault, StéphaneMaksymiuk, Andrew W.Archambault, VincentMalapelle, UmbertoArciello, AngelaMalarkannan, SubramaniamArcone, RosariaMalejko, JulitaArdalan, MaryamMałgorzata, KowalskaArdejani, MaziarMalinowska, ElzbietaArenas, JesúsMallela, Shamroop KumarArenas-Mena, CesarMallipeddi, Prema LathaArese, MarziaMalone, JacobArguelles, SandroMalys, NaglisArif, TasleemMambu, LengoArimura, ShinichiMammadova-Bach, ElminaArizza, VincenzoMandraffino, GiuseppeArmanini, DecioMangiagalli, MarcoArnaboldi, PaulMangino, GiorgioArriagada, CésarMangogna, AlessandroArrizon, JavierMangoni, Maria LuisaArthur, HelenMani, AlirezaArvidsson, Per I.Maniati, EleniAsa’ad, FarahMankin, AlexanderAsada, HidetsuguMann, FrancisAsada, KenMänner, JörgAsamizu, ShumpeiMansour, SalahAschtgen, Marie-stephanieMansuri, ShahidAsensio, Juan LuisManta, StellaAshaie, MaeirahMantere, OutiAshihara, EishiManzo-Merino, JoaquinAshour, Mohamed LotfyManzoni, Olivier J.Ashton, Nicholas W.Mao, XiaoboAssaf, Khaleel IbrahimMao, XumingAszodi, AttilaMarabello, DomenicaAtaide, SandroMaraia, RichardAtanasova, NinaMarasek-Ciolakowska, AgnieszkaAtreya, Chintamani D.Marazuela, MonicaAttanzio, AlessandroMarc, GabrielAttili, Anna RitaMarchand, ChristopheAudrito, ValentinaMarchetti, DarioAugustine, JosyMarchev, AndreyAuriemma, GiuliaMarchini, CristinaAutry, JosephMarcinkowska, EwaAvdonin, Pavel V.Marco-Contelles, Jose LuisAviles, Francesc XavierMarczyk, MichalAviñó, AnnaMaréchal, AlexandreAyabe, TatsuhiroMaresca, MarcAyonrinde, Oyekoya T.Marginǎ, DenisaAyyoob, MuhammadMargiotta, AzzurraAzarnia Tehran, DomenicoMargis, RogerioAzarov, Jan E.Marí, MontserratAzevedo, JorgeMaria Carmela, Bonaccorsi Di PattiAzzini, ElenaMarietta, FodorBabichev, SergiiMarinello, FrancescoBabu, Gopal J.Marino, AngelaBąchor, RemigiuszMarino, ValerioBacik, John-PaulMarkova-Car, Elitza P.Back, KyoungwhanMarkowicz-Piasecka, MagdalenaBadea, MihaelaMarkowska-Daniel, IwonaBade-Doeding, ChristinaMarkwardt, FritzBadu-Tawiah, AbrahamMarlowe, TimothyBaggiani, ClaudioMarmagne, AnneBaglietto-Vargas, DavidMaroni, PaolaBagnard, DominiqueMaroteaux, LucBagoly, ZsuzsaMarques, Alexandra P.Bahuguna, AshutoshMarques, MarianaBai, ShuhuaMarquez, EdgarBaiesi, MarcoMarrs, James A.Bailey, Charles GMarsche, GuntherBailly, BenjaminMarshall, CraigBajoghli, BaubakMartelli, FrancescoBajusz, DávidMartens, GerardBakaeva, ZandaMartí, SergioBakhti, MostafaMartin, AndreasBakillah, AhmedMartin, Andrew C.R.Balan, PrabhuMartin, FranckBalcerczak, EwaMartín, Juan FranciscoBalcerek, MariaMartin, PamelaBaldo, GuilhermeMartinez, AgustinBalestra, CostantinoMartinez, AnaBall, Zachary T.Martínez, ConstantinoBaltina, LidiaMartínez, SoniaBalzano, TizianoMartínez-Banaclocha, Marcos ArturoBan, BhupalMartínez-Campa, CarlosBanerjee, SantanuMartínez-Force, E.Banfalvi, ZsofiaMartinez-Gonzalez, Luis J.Bang, John J.Martínez-Martínez, IreneBaniene, RasaMartínez-Salas, EncarnaciónBanito, AnaMartínez-Yamout, Maria A.Bañuls, Maria JoseMartínková, LudmilaBao, JinkuMartín-Montalvo, AlejandroBao, Jun-XiangMartinou, Jean-ClaudeBaptista, Mafalda S.Martins, NataliaBarabutis, NektariosMartins, Pedro M.Baraldi, ElenaMartín-Ventura, José LuisBaranowska-Kuczko, MartaMaruca, AnnalisaBarault, LudovicMaruška, AudriusBarbagallo, DavideMarutescu, Luminita GabrielaBarbosa, ArmenioMarverti, GaetanoBarchetta, IlariaMary, DidierBarderas, RodrigoMassa, AnnamariaBärebring, LinneaMassaro, MarinaBarfield, RobynMast, YvonneBarker, TylerMastanjević, KristinaBarnea, Eytan RMastinu, AndreaBarnett, SusanMastoraki, AikateriniBar-Or, DavidMastracci, TeresaBarranco, IsabelMastrangelo, Anna M.Barreto, GuillermoMastrotto, FrancescaBarrett, JamesMasuda, KiyoshiBarros, Marcelo PaesMasuyer, GeoffreyBarshtein, GregoryMatafome, Paulo N.Barta, CsengeleMatás-Parra, CarmenBartolazzi, ArmandoMateo-Bonmatí, EduardoBartolini, ManuelaMatesic, LidiaBartolini, PaoloMateus, Maria L.Bartolotti, MassimoMatichenkov, Vladimir V.Barton, Matthew JMatilla, MiguelBartova, EvaMatkowski, AdamBartuzi, DamianMato, EugeniaBarvik, IvanMato, SusanaBarygin, Oleg I.Matsuda, HisashiBasak, DebasishMatsufuji, HiroshiBasilio Heredia, JoseMatsugaki, AiraBasini, GiuseppinaMatsukawa, NoriyukiBasith, ShaherinMatsumoto, TaroBassanini, IvanMatsuura, KazunoriBasso, DanielaMatsuzaki, JuntaroBassolino, LauraMatsuzaki, KeiichiBateman, AndrewMattei, BenedettaBatissoco, Ana C.Mattei, CésarBatsché, EricMattinen, Maija-LiisaBattistelli, MichelaMattingly, Raymond R.Baucum, Anthony J.Mattoo, Autar K.Baudier, JacquesMattos, CarlaBauer, JohannMatucci, RosannaBauer, StefanieMatulić, MajaBaumgartner, UlrichMatuszewska, AnnaBautista, MartaMatwijczuk, ArkadiuszBax, Bridget E.Matysiak, JanBayascas, Jose RMatysiak, JoannaBayer, PeterMatziari, MagdaliniBayfield, MarkMauceri, RodolfoBeale, DavidMavon, AlainBeamer, Lesa J.Mavragani, ClioBecciolini, AndreaMavri, JanezBecker, ThomasMavrogonatou, EleniBeckett, DorothyMayan, Maria D.Bednarek, PiotrMayeda, AkilaBednárová, LucieMayer, Thomas U.Beerhues, LudgerMayo, Juan CarlosBegemann, GerritMayo, KevinBeharry, Kay D.Mazerbourg, SabineBeilby, MaryMazur, MarcelinaBekatorou, ArgyroMazzanti, AndreaBelayew, AlexandraMazzulli, JoeBełdowski, PiotrMccully, Kilmer S.Belfiore, AntoninoMcDowell, Colleen M.Belin De Chantemèle, Eric J.McGavin, Martin J.Bellier, Jean PierreMcGuckin, MichaelBelluzzi, ElisaMcHugh, Kevin J.Beltowski, JerzyMcKay, TinaBelyi, YuryMcKinnie, ShaunBenabdellah, KarimMcKinstry, Karl KaiBenarba, BachirMd Badrul, AlamBenard, GiovanniMears, JasonBendas, GerdMeccariello, RosariaBenedec, DanielaMedici, SerenellaBenevolenskaya, ElizavetaMedina-Rodríguez, Eva M.Benfenati, ValentinaMedraño-Fernandez, IriaBenistant, ChristineMedvedev, AlexeiBennett, James P.Meerson, AriBennett, Robert G.Megyeri, KlaraBenohoud, MeryemMehta, Gaurav A.Berdis, Anthony J.Meinander, AnnikaBerenguer, JoseMeiners, SilkeBerenson, JamesMeldolesi, JacopoBerenyiova, AndreaMellata, MelhaBerger, TrishMemo, MaurizioBergler, HelmutMenanteau–Ledouble, SimonBerliner, Lawrence J.Mendez-Cuadro, DaríoBernabéu, CarmeloMendonca, PatriciaBernal, Freddy AlexanderMendoza, ArtemioBernardes-Génisson, VaniaMenegon, AndreaBernardi, TatianaMENEGUZZO, FRANCESCOBernstein, NiritMenendez, DanielBerthold-Pluta, AnnaMeneses, CarlosBerthoud, Viviana MMenezes, Irwin Rose AlencarBerti, FedericoMennerich, DanielaBertin, Matthew J.Merimi, MakramBertolotti, MarcoMerino Peláez, GraciaBertrand, CzarnyMertens, PeterBesenfelder, Urban H.Meškys, RolandasBessho, TadayoshiMeslet-Cladiere, LaurenceBeuning, PennyMessina, SamanthaBeverly, Levi J.Messner, BarbaraBevilacqua, VitoantonioMeštrović, TomislavBeyer, Eric C.Metzinger, LaurentBhandari, Manohar PrasadMeyer, Thomas J.Bharadwaj, ShivMézes, MiklósBhat, KamakotiMezheyeuski, ArturBhattacharya, AbhisekMezquita, CristóbalBhattacharya, SupriyoMichaud, PhilippeBhoobalan-Chitty, YuvarajMichlewski, GracjanBiagi, MarcoMiddleton, AdamBianchi, VittorioMidgley, AdamBielczyk-Maczyńska, EwaMignatti, PaoloBiener, MoritzMiguel-Hidalgo, Jose JavierBignon, ChristopheMika, DelphineBijak, MichałMikolajczak, PrzemyslawBikiaris, DimitriosMikros, EmmanuelBilal, MuhammadMilani, AdelaideBileck, AndreaMilanovic, DesankaBills, Gerald F.Milcarek, ChristineBilska-Wilkosz, AnnaMileva, MilkaBilski, JanMillán-Linares, María Del CarmenBiondi, BarbaraMiller, CharlesBirková, AnnaMillet, ÓscarBischof, JoachimMinato, Ken-ichiroBischof, JohannesMiniero, Daniela ValeriaBishop, RogerMinucci, SergioBisig, ChristophMinutolo, FilippoBisson, AlexandreMiosge, NicolaiBitto, AlessandraMir, RicardoBizon, KatarzynaMiranda, CristobalBlack, Esther P.Miranda, HugoBlakely, William F.Mirkov, IvanaBlanco Fernandez, MatiasMishra, AwdheshBlanco, EduardoMishra, Geetesh K.Blanco, PilarMishra, ManishBlanquart, ChristopheMishra, NigamBlasiak, JanuszMishra, Pawan KumarBlumenschein, TharinMishra, Sandeep KumarBoccaccini, AlessandraMiskulin, MajaBoccuni, FabioMitchell A, SullivanBochenkova, Anastasia V.Mitchell, Jennifer A.Bocian, SzymonMitchell, Molly E.Bock, FlorianMitchell, ToddBoer, Vincent C. J. DeMitola, StefaniaBoffano, PaoloMitraki, AnnaBogdanov, Alexey A.Miwa, Julie M.Böhmer, FrankMiyakawa, IsamuBohn, ErwinMiyamoto, YoichiBojarová, PavlaMiyata, YasuyoshiBojarska, JoannaMiyazaki, IkukoBojarski, AndrzejMiyoshi, EijiBoland, SebastianMiyoshi, Shin-ichiBoldingh, HelenMizrahi, BoazBolotin, MoniqueMizumoto, ShujiBolt, Edward L.Mladenović, MilanBölükbas, DenizMo, FeiBomken, SimonMobbili, GiovannaBoncela, JoannaMocquet, VincentBonciu, ElenaMofrad, MohammadBonet, Esther UdinaMohamed, Azza H.Bonfiglio, RitaMohamed, Junaith S.Bonfio, ClaudiaMohammed, AltafBonifacio, Maria AddolorataMoiseyev, GennadiyBonomini, MarioMoldovan, BiancaBontems, FrançoisMolina Salinas, Gloria MaríaBonuccelli, GloriaMolina, LázaroBooth, StephanieMolinier, JeanBorges, RicardoMollen, Kevin P.Borgnia, Mario J.Molnár, ElekBoris, PejinMonari, AntonioBoroń, DariuszMondal, GoutamBoronat, AnnaMondal, SudipBorota, AnaMondal, Utpal KumarBorovac, Josip A.Mondeshki, MihailBorse, VikrantMonné, MagnusBorst, Jan WillemMonsivais, DianaBorysiak, SlawomirMontag, DirkBosak, AnitaMontagne, KevinBose, DebojitMontana, Angel M.Bossche, Jan Van DenMonte Lara, Mª ConcepciónBossis, GuillaumeMonti, Maria ChiaraBosso, AndreaMontpetit, BenBottani, EmanuelaMoore, Ronald BBottini, MassimoMoore, StuartBougard, DaisyMorak-Młodawska, BeataBouitbir, JamalMorales, LudisBounaceur, RodaMorales-Morales, DavidBourdon, EmmanuelMorante, KoldoBourgault, SteveMoreira, Daniel CarneiroBourgougnon, NathalieMoreira, Manuela M.Bouschet, TristanMoreira, PaulaBoutelle, Martyn G.Morello, RoyBovin, NicolaiMoreno Amador, María LourdesBovolin, PatriziaMoreno Gonzalez, InesBowen, Mark E.Moreno, EstherBowman, DarylMoretti, FabiolaBoysen, ReinhardMorgan-Bathke, MariaBozzaro, SalvatoreMorgat, ClémentBracco, EnricoMorgunov, IgorBradbury, AllisonMoricz, AgnesBrahmachari, SauravMoriishi, KohjiBraicu, CorneliaMorikawa, SatoruBrand, IzabellaMorimoto, Yusuke V.Brandenburg, Lars OveMorinobu, AkioBrandl, Christopher J.Morita, TomotakeBrannick, Erin M.Moriya, ManabuBrasó-Maristany, FaraMoriyama, MarikoBraun, RalfMoriyama, TatsuyaBravo-Sanchez, M. G.Morla, ShravanBreckpot, KarineMorleo, ManuelaBregier-Jarzębowska, RomualdaMorley, Barbara J.Brem, JurgenMoro, EnricoBren, UrbanMoro, LoredanaBrendel, JohannesMorocho, VladimirBrennan, CarolineMorozova-Roche, LudmillaBrenowitz, MichaelMorris, AndrewBresolí-Obach, RogerMorrison, Keith D.Brestic, MarianMortara, LorenzoBreuzard, GillesMoseley, HunterBriedé, JaccoMoses, TessaBriers, YvesMoskalewicz, TomaszBrighenti, VirginiaMoskot, MartaBrígido, ClarisseMoss, MarkBrindisi, MatteoMotin, VladimirBriza, PeterMotohashi, KenBroda, Małgorzata A.Mott, HelenBrodolin, Konstantin L.Motti, Maria LetiziaBrogi, SimoneMouga, TeresaBromage, ErinMounien, LourdesBronstein, JeffMoya-Ramírez, IgnacioBrosius, JürgenMravljak, JanezBrown, Ashley N.Mrozek-Wilczkiewicz, AnnaBrown, LindsayMu, YunxiangBrown, Paul BMuench, StephenBrown, Robert J.Mugisho, OdunayoBrozmanová, MarianaMukaida, NaofumiBrozyna, AnnaMukhopadhyay, SumanBru, SamuelMulenga, AlbertBruchmann, SebastianMüller, ChristophBrückner, AdrianMüller, NorbertBruins, Jantinus H.Müller-Taubenberger, AnnetteBrull, AstridMulligan, Vikram K.Brunetti, AngelaMulloy, BarbaraBrunetti, LuigiMulo, PaulaBrunetti, OronzoMummidi, SrinivasBruno, StefaniaMumoli, NicolaBruzzone, SantinaMuñoz Caffarel, Maríabrzoska, malgorzata michalinaMuñoz, FranciscoBubici, GiovanniMunteanu, Florentina-DanielaBuchberger, AlexanderMunusamy, ElangoBudzisz, ElżbietaMurakami, HironobuBueno-Silva, BrunoMurakami, Katsuhiko S.Bukhari, Marwan A.S.Murakami, MakotoBukrinsky, MichaelMurakami, MasaakiBulati, MatteoMuramoto, KojiBulc, MichałMurase, SachikoBunea, AndreaMurata, ShigeoBungau, SimonaMurkin, AndrewBungau, Simona GabrielaMuronetz, VladimirBungert, JörgMuroya, SusumuBunik, VictoriaMurphy, Caroline SBuonaguro, Elisabetta FilomenaMurray Stewart, TracyBurbage, MarianneMurthy, VinuthaaBurch-Smith, TessaMusalgaonkar, SharmishthaBurcul, FrankoMuscara, Marcelo N.Burger, Michael CMusco, GiovannaBurgos, HéctorMustafa, Ahmed M.Burguete, Maria ConsueloMuszyński, SiemowitBurke, Andrew RobertMutti, LucianoBurkhead, JasonMuyldermans, SergeBürklein, SebastianMyöhänen, Timo T.Burlacu, AlexandruMythreye, KarthikeyanBurpo, Fred J.Na, Dong HeeBurton, Zachary F.Na, MinKyunBurvenich, IngridNaafs, BernardBusath, David D.Nadǎş, George CosminBusch, AlbertNaderi, MohammadBussmann, Rainer W.Nadiminty, NagalakshmiBustaffa, ElisaNagafuchi, YasuoBustos, Diego MartinNagaoka, IsaoButcher, AdrianNagaraj, ViniBuzoianu, AncaNagendran, JeevanBydlowski, Sérgio PauloNaggi, AnnamariaByeon, HaewonNagy, VeronikaByrne, BernadetteNagyova, EvaByrne, Mark E.Naim, HassanBystrom, JonasNair, SreenathBystrowska, BeataNajdekr, LukášByun, SanguineNakagaki, TakehiroCabaro, SerenaNakagawa, PabloCacanyiova, SonaNakahata, MasakiCaceres, JavierNakajima, KeiCádiz-Gurrea, María De La LuzNakamaru-Ogiso, EikoCafiero, MauricioNakashima, SouichiCagnin, StefanoNakata, RiekoCai, KaiNakatani, YosukeCairrão, ElisaNakatsu, KanjiCAKIR, BirsenNakazawa, YasumotoCal, SantiagoNalepa, IrenaCalarco, AnnaNamasivayam, VigneshwaranCaldara, MarinaNamkung, WanCaldwell, GaryNance, ElizabethCallaghan, DavidNanda, Sitansu SekharCaltabiano, RosarioNanduri, RavikanthCalvano, Cosima D.Nanoff, ChristianCalvo Guirado, José LuisNarayanan, S. PriyaCameron, AngusNardo, LucaCameron, DonaldNaro, FabioCameron, SimonNarra, Hema PrasadCametti, CesareNaryzhny, Stanislav NCamila, Hochman-MendezNash, Michael A.Camire, MaryNassir, FatihaCampbell, GraceNatalello, AntoninoCampilho, AnaNatalicchio, AnnalisaCampomenosi, PaolaNauli, SuryaCamporesi, Enrico M.Naumowicz, MonikaCampos Rosa, Joaquín MaríaNavarro, Juan AntonioCanals, IsaacNaya, FranciscoCanchaya, CarlosNazar, Ross N.Candéias, SergeNazzaro, FilomenaCandela, Diaz-CanestroNeeb, AntjeCanela, Enric I.Neginskaya, Maria A.Cannas, AngelaNémeth, BalázsCanonico, BarbaraNesmelova, IrinaCanonico, LauraNesterkina, MariiaCao, JianNeubauer, HeidiCao, WeiNeustupa, JiriCao, XianNeu-Yilik, GabrieleCapasso, RaffaeleNeves, JoãoCapece, AngelaNewman, DavidCaplan, AllanNewton, PhillipCapozzi, VittorioNg, Chen SiangCappello, PaolaNg, Jason Liang PinCaprio, MassimilianoNg, Roy Chun-LaamCapuano, VittorioNgara, RudoCaputa, MichałNgezahayo, AnacletCaputo, LuciaNguyen, Van KhanhCarazo, AlejandroNicaud, Jean-MarcCarboni, LuciaNicolle, RemyCardellicchio, CosimoNicosia, AldoCardenas, MaritéNiculescu, LoredanCardona, FernandoNiczyporyk, Jowita SamantaCardoso, Susana M.Niederreither, KarenCaretta, AntonioNieland, John Dirk VestergaardCariani, ElisabettaNielsen, Sofie V.Carlisi, DanielaNiemann, Hartmut H.Carlone, Robert L.Niero, GiovanniCarlos, Sao-JoseNikitovic, DraganaCarman, George M.Nikolaivits, EfstratiosCarmichael, Gordon GNikolich, MikeljonCarmona-Saez, PedroNikolova, MariaCarnevali, DavideNilsen, HildeCarone, Benjamin R.Nilsson, AkeCarradori, SimoneNinfali, PaolinoCarrieri, AntonioNioi, ClaudiaCarrizzo, AlbinoNirala, Niraj K.Carro, EvaNirmala, JayaveeramuthuCarter, WayneNirody, Jasmine A.Carugo, OlivieroNishi, KosukeCarullo, GabrieleNishimiya, YoshiyukiCaruntu, ConstantinNishitsuji, KazuchikaCaruso, FrancescoNisticò, PaolaCarvalho Pimenta, DanielNitulescu, George MihaiCarvalho, António PauloNociari, MarceloCarver, John A.Nohe, AnjaCasampai, AntalNomura, MasatoshiCasapullo, AgostinoNonaka, TaichiroCasar, BertaNonogaki, KatsunoriCasas, Rosa M.Nord, AshleyCasella, LuigiNordmark, GunnelCasillas, SoniaNorihiko, NaritaCaspari, ThomasNotarbartolo, MonicaCassinelli, GiulianaNotarstefano, ValentinaCastaing, BertrandNovak Kujundžić, RenataCastiglia, DanielaNovinec, MarkoCastilla, JoaquinNR, ParineCastillo, AntonioNucci, NathanielCastillo, FranciscoNunes, AlexandraCastillo-Tong, Dan CacsireNunez, CesarCaston, José RNunzio, VicarioCastresana, Javier S.Nurunnabi, MdCastro, Mariana S.Nussinov, RuthCASTRONUOVO, DonatoNuttall, Patricia ACaswell, Clayton C.Nycz, JacekCatalan, MarceloNyström, AlexanderCatalani, ElisabettaO’Leary, SeánCatanzaro, ElenaObeng, Esther A.Catanzaro, GiuseppinaOberemok, Volodymyr V.Cathy, JacksonOberle, Doris F.Caughey, ByronObermayer, BenediktCavaliere, GinaOchi, ShinichiroCavallo, GisellaOchieng, JosiahCavazza, ChristineOchsenreither, KatrinCaviglia, Gian PaoloOdland, JonCavoretto, PaoloOelschlaeger, PeterCaza, TiffanyOfir, RivkaCeci, Luigi R.Ogawa, TakahiroCelebioglu, AsliOh, Deog-HwanCembrowska-Lech, DanutaOh, Jae-MinCendron, LauraOh, JoonseokCerdeira, Cláudio DanielOh, Seung JaCereda, MatteoOhki, RiekoCerezo, JavierOhko, KentaroČerná, MarieOhlenschläger, OliverČerný, JiříOhta, KiminoriCeron-Carrasco, JoseOhta, NobuoCerrito, Maria GraziaOishi, KimihikoCervellati, FrancoOkada, MotohiroCervera, AnaOkae, HiroakiCervetto, ChiaraOkamura, RyosukeCéspedes, MaríaOkolicsanyi, Rachel KCevenini, ArmandoOku, MasahideChachami, GeorgiaOkuda, KazuhideChadwick, RuthOlejar, Kenneth J.Chagas, Juana C. CaririOlennikov, DaniilChaimbault, PatrickOliva, RominaChakrabarti, SutapaOliveira, AnaChambers, Jeremy W.Oliveira, Guedmiller S.Chan, Yau KeiOliveira, SabrinaChan, Yin-ChingOliviero, FrancescaChand, Kirat K.Ollero, MarioChandrasekaran, Arun RichardOlofsson, MalinChang, Chia-cheOlsen, BirgitteChang, FeiOlsen, Lars FolkeChang, Kee-lungOlson, EricChang, Ling-ChuOlson, KennethChang, Long-SenOlsson, StefanChang, Margaret Dah-tsyrOlszewska, MartaChang, Te-ShengOltean, SebastianChang, Wen-ChiOluwadare, OluwatosinChang, Yuan-YenOmar, Syed HarisChang, Yu-WeiOndrias, KarolChao, Yun-YangOnduka, ToshimitsuChapoval, Svetlana P.Ong, QunxiangCharbonneau, MichelOniga, IlioaraCharreau, BeatriceOniga, Smaranda DafinaChatterjee, MadhumitaOnoyama, IchiroChaudhari, KiranOpaliński, ŁukaszChaudhry, BillOprea, Corneliu IoanChaudhry, RasulOpricǎ, LăcrămioaraChauhan, NeerajOrdaz-Ortiz, José JuanChaumont Dubel, SeverineOrenes Piñero, EstebanChaurasia, A. K.Oriá, Reinaldo B.Chauvot De Beauchene, IsaureOrian, LauraChavez-Santoscoy, Rocio AlejandraOriuchi, NoboruChazin, WalterOrlando, GiustinoChecconi, PaolaOrłowski, MarekCheccucci, AliceOrrú, ChristinaCheemarla, Nagarjuna ReddyOrtega-Villaizan, Maria Del MarCheignon, ClémenceOsadnik, TadeuszCheloha, RossOsmanagic-Myers, SelmaChen, Chin-ChuOsmulski, Pawel A.Chen, Chung-MingOstacolo, CarmineChen, CortinaOstash, BohdanChen, HuOstoa-Saloma, PedroChen, Jen-TsungOstrovsky, OlgaChen, Ji-YihOta, MotonoriChen, Lih-GeengOtt, MariaChen, MuyuanOugham, HelenChen, PanOury, BrunoChen, Po-YuanOvádi, JuditChen, QiOvermyer, KatherineChen, QiushengOves-Costales, DanielChen, QunOwen, ShawnChen, Ray-BingOzaki, ToshinoriChen, Rong-JaneOzawa, ShogoChen, ShuaiOzen, MehmetChen, VincentOzimek, EwaChen, XingqiPaavola, JereChen, XixianPace, LorettaChen, YaPacheco, NeithChen, Yann-JangPacholski, AndreasChen, Yi-HauPaci, MaurizioChen, YingPaczesny, JanChen, YuPaduszynska, MalgorzataChen, ZhaoPagès, VincentCheng, Chia-HsiungPaidi, Santosh KumarCheng, Chi-LienPainter, HeatherCheng, Ching-YiPakhomova, SvetlanaCheng, HailiPalage, MarianaCheng, Hong ShengPalanisamy, ArulselvanCheng, Juei-tangPalanisamy, Uma DeviCheng, Wei-chiehPALAVAI, SRIPAL REDDYCheng, XiaomingPalenzuela López, José AntonioCheng, Yuan-BinPalermo, GiuliaChengwei, YangPalini, SimoneChen-Hui, ChenPallàs Lliberia, MercèChérel, IsabellePalma, MiguelChern, EdwardPalmer, AlanChernikov, OlegPalmeri, RosaChernova, TatianaPalomo, ValleCheung, David L.Palumaa, PeepCheungpasitporn, WisitPalyulin, VladimirChew, Nick Guan PinPan, CuipingChi, Ya-HuiPan, JiayiChiang, Hung-LungPan, PingyueChiappini, FranckPanaro, Maria AntoniettaChiariotti, LorenzoPanatala, RadhakrishnanChibbar, RavindraPancsa, RitaChien, Chih-YenPanda, RakhiChien, PeterPandey, Avinash ChandraChieppa, MarcelloPandey, ChandanaChin, Kok YongPang, Myung-GeolChin, Young-WonPannek, JürgenChini, AndreaPantano, LorenaChino, MarcoPanzella, LuciaChirikova, Nadezhda K.Paoli, PaoloChirumbolo, SalvatorePapaioannou, EmmanouilChittori, SagarPapathanasiou, FokionChiummiento, LuciaPapini, AlessioChlubek, DariuszPapp, GáborChmurzyńska, AgataParchi, PieroCho, HyosunPardo, FatimaCho, Jung SookPardo, MartaCho, NamkiParenti, CarmelaCho, YongyeonParikesit, Arli AdityaChoi, Hyung-KyoonParis, JuanChoi, SangchunParisi, LudovicaChoi, Yoon KyungPark, DaehoChoromanska, AnnaPark, Edwards A.Chou, YachangPark, Jong KookChoudhury, Swarup RoyPark, Seong-CheolChow, Christine S.Park, WansuChow, Kwok-FanPark, Woo JaeChowdhury, Ezharul HoquePark, YoonseongChowdhury, FarhanParkar, Shanthi G.Christodoulou, Maria-IoannaParkkita, SeppoChristoforides, EliasParmeggiani, FabioChuang, Hung-YiParnetti, LucillaChubiz, LonParra, MargaritaChueh, Pin JuParrino, VincenzoCHUI, Kwok TaiPârvu, Alina ElenaChun, SungkunPascal, Steven M.Chung, SungjinPasciu, ValeriaChung, Won-YoonPashov, AnastasCianciosi, DanilaPasqualini, FrancescoCiandrini, LucaPasquinelli, GianandreaCiani, MaurizioPastores, Gregory M.Ciaudo, ConstancePašukonienė, VitaCicala, CarlaPatelski, PiotrCichero, ElenaPatra, Jayanta KumarCieplak, MarekPatro-Małysza, JolantaCiesielski, SlawomirPatterson, JenniferCifuentes, DanielPaudel, IndiraCilla, AntonioPaul, AnanyaCimadevilla, JosePaul, SudipCinar, BekirPautz, AndreaCindrić, MarioPavlik, Edward J.Cioanca, OanaPavlin, MaticCiornea, Elena TodirascuPawlik, AnnaCipolletti, ManuelaPearce, MargaretCiregia, FedericaPeart, JasonCirrincione, GirolamoPec, MartinCitti, CinziaPecic, StevanClara, MattuPecqueur, ClaireClarke, Anthony J.Pedemonte, NicolettaClarke, ChirstopherPedersen, Per AmstrupClarke, JonathanPedeux, RémyClarke, StephenPedraza Chaverri, JoséClaus, HaraldPedrero, MariaClemens, RogerPedroso, João AlfredoClementi, CatiaPegan, Scott D.Clichici, SimonaPeiser, MatthiasCloninger, MaryPeitzsch, ClaudiaClose, PierrePeixoto, PaulCocco, TizianaPelagalli, AlessandraCockburn, DarrellPellegrini, MarikaCoelho-Silva, JuanPellegrino, MarshaCoenen, Marieke J.H.Pelzer, EliseCoetzee, William A.Pemp, BertholdCoffman, LanPeña Vega, FernandoCohen, SethPeña-Espinoza, MiguelCoin, IrenePeña-Rodríguez, LMCoisson, Jean DanielPenas, ClaraColeine, ClaudiaPence, BrandtColige, AlainPeng, Robert Y.Collavin, LicioPeng, WenjingColliec-Jouault, SylviaPeng, XuCollins, John D.Pentimalli, FrancescaColomba, PaoloPenzo, MariannaColomina, Maria TeresaPeptu, CristianColone, MarisaPerdomo, JoseColpitts, Che C.Perego, PaolaColston, Timothy J.Pereira, CláudiaColuccia, AntonioPereira, FlorbelaColucci-D’Amato, LucaPereira-Leite, CatarinaColussi, Gian LucaPerestrelo, Rosa Maria De SáColussi, SilviaPérez - Amodio, SoledadComai, StefanoPérez De Vega, María JesúsComoy, EmmanuelPerez-Hernandez, JavierConflitti, PaoloPerez-Mendez, OscarConrad, David MPergolizzi, SimonaConstantinescu-Aruxandei, DianaPerinelli, Diego RomanoConsumi, MarcoPerišić, OgnjenConte, Maria SasiPeriyannan, SambasivamConti, BicePerkins, ArdenContreras-Cornejo, Hexon AngelPermyakov, EugeneConvertini, PaoloPernisová, MarkétaConway, EdwardPernodet, Jean LucCooke, AmyPero, RaffaelaCopic, AlenkaPerra, AndreaCopolovici, Dana MariaPerreault, MelissaCopolovici, LucianPerricone, CarloCorazzari, MarcoPerrucci, StefaniaCorbeil, DenisPers, Yves-MarieCordeiro, YraimaPerugino, GiuseppeCordero, Chiara EmiliaPeruzynska, MagdalenaCordonnier, AgnèsPestov, DimitriCornejo, AlfonsoPetan, ToniCornelison, Christopher T.Petca, RǎzvanCorr, SinéadPeter, Emanuel KarlCorsaro, AlessandroPeters, ChristianCorzo-León, Dora E.Petersen, Robert B.Cosentino, Nicola M.Petersen, SveaCosta De Camargo, AdrianoPeterson, JuliaCosta, JoanaPeterson, LarrynCosta, Marina C.Peterson-Hiller, AmieCosta, RaquelPetit, Patrice X.Costa, RobertoPetrov, Alexey M.Cote, ChristopherPetter, MichaelaCotignola, JavierPezzolesi, LauraCouillaud, FranckPfeffer, Lawrence M.Courtois, GillesPfister, Astrid S.Coyle, Patricia K.Pfister, BarbaraCozza, GiorgioPhelix, Clyde F.Crehuet, RamonPhilibert, DanielleCrépeaux, GuillemettePhilpott, CarolineCrespo-Hernández, CarlosPia̧tek, Rafał J.Crisafulli, GiovanniPiato, AngeloCrisponi, GuidoPiccialli, GennaroCristofori-Armstrong, BenPiccionello, Antonio PalumboCrosas, BernatPiccoli, MarcoCrosson, SeanPick, ElahCrowder, MichaelPidoux, GuillaumeCruz, CarlaPiedras, PedroCruz, RebecaPiehler, JacobCsapo, EditPieretti, StefanoCséplő, ÁgnesPierri, Ciro LeonardoCsupor, DezsőPierzchalski, PiotrCubellis, MariaPiestrzyńska-Kajtoch, AgataCubero, Francisco JavierPietropaolo, AdrianaCuff, SimonePievani, AliceCui, HaissiPignitter, MarcCui, HuaqingPigossi, Suzane CristinaCui, HuiPilling, DarrellCui, QingbinPineda, José R.Cui, XiaoyingPineda, Jose RamonCummings, BrianPiñeiro, AngelCunha, Rodrigo A.Pinelis, V. G.Cunningham, Christopher W.Ping, Yueh-HsinCuppoletti, JohnPinter, TylerCuri-Borda, Cecilia K.Pinteus, SuseteCurnow, PaulPinto, SandraCurtin, Nicola JPintus, FrancescaCutignano, AdelePintus, GianfrancoCuya, Ricardo TeobaldoPiomboni, PaolaCuzzolin, AlbertoPiotrowicz-Cieślak, AgnieszkaCzarnocki, ZbigniewPiras, Anna MariaCzechowska, JoannaPires, Bruno R. B.Czernel, GrzegorzPires, CarlaCzogalla, AleksanderPirola, LucianoCzumaj, AleksandraPirooznia, MehdiCzyż, KatarzynaPishchalnikov, Roman Y.Czyżnikowska, ŻanetaPissas, GeorgiosD. Bortner, CarlPistarà, VenerandoD’Amelio, RaffaelePistocchi, Anna SilviaD’Anneo, AntonellaPitingolo, GabrieleD’Arcy, PadraigPitts, MatthewD’arcy, PádraigPiva, TerrenceD’Elia, DomenicaPiya, SarbottamD’Errico, StefanoPizoń, MagdalenaD’Onofrio, NunziaPizzo, ElioD’Orazi, GabriellaPlant, LeighDa Silva, Gabriela JorgePlatta, HaraldDaberdaku, SebastianPlatts, JamesDaci, ArmondPlaza-Zabala, AinhoaDadakova, EvaPlenis, AlinaDaehn, IlsePleschka, StephanDai, YuminPlíhal, OndřejDal Prá, IlariaPluta, RyszardDalby, Kevin N.Podlipnik, ČrtomirDall’Acqua, StefanoPodobnik, MarjetkaDamiano, FabrizioPoeggeler, BurkhardDanac, RamonaPohl, EhmkeDanesi, FrancescaPoinsot, VerenaDangles, OlivierPojo, MartaDaniellou, RichardPóka, RóbertDanilo, CucchiPolacek, NorbertDaragó, AdamPolčic, PeterDarenskaya, M. A.Polhemus, DavidDarling, Andrea L.Polit, AgnieszkaDaroch, MaurycyPollastri, GianlucaDas, AninditaPollice, AlessandraDas, JoydipPoloni, Tino EmanueleDas, ViswanathPomari, ElenaDash, BirajaPompilio, GiulioDashti, YousefPonce-Vargas, MiguelDaughdrill, Gary W.Ponnalagu, DevasenaDavies, ChristopherPooler, DarcyDavies, LindsayPoon, GregoryDavignon, Jean-lucPop, Horia FDavis, FredPope, ChadDavuluri, GangaraoPopescu, Mihaela R.Dawood, Mahmoud A.O.Popgeorgiev, NikolayDay, Catherine L.Popielarska-Konieczna, MarzenaDe Angelis, AnnaPoplawski, TomaszDe Araujo, ElvinPorcari, Andréia De MeloDe Bellis, LuigiPorrini, VanessaDe Craene, Johan-OwenPotanin, Andrei A.De Crécy-Lagard, ValériePotočnik, UrošDe Deurwaerdere, PhilippePotočnjak, IvaDe Falco, EnricaPotrich, CristinaDe Feo, VincenzoPottosin, IgorDe Giorgi, Maria LuisaPoulin, RemingtonDe Guia, Roldan M.Poupot, RemyDe La Luz Garcia-Hernandez, MariaPourcet, BenoîtDe La Vega, LaureanoPourzand, ChararehDe Lago, EvaPowell, Folami LamokeDe Ligt, JoepPowell, GregoryDe Los Rios, CristobalPower, DavidDe Luca, AnastasiaPowers, RachelDe Marco, ArioPracharova, JitkaDe Masi, LuigiPramanik, AvijitDe Molina, Ana RamírezPrasad, RamDe Moreno, APrasanna, PradeepDe Paepe, BoelPraud, ChristopheDe Pinto, VitoPreiner, DarkoDe Rasmo, DomenicoPreiss, ThomasDe Rosa, SalvatorePrelipceanu, MariusDe Vega, AntonioPrester, LjerkaDe Vita, DanielaPreti, DeliaDeAngelis, PaulPriault, MurielDel Re, MarziaPrieto-Garcia, Jose M.Delbarre Ladrat, ChristinePrimožič, InesDell’Orco, DanielePrinz, WilliamDell’Oste, ValentinaPritzker, KennethDella Sala, GerardoPritzkow, SandraDelogu, GiovannaProcházková, JiřinaDempfle, AstridProctor, Spencer D.Dempsey, Christopher E.Prudent, JulienDeng, HaiPrusty, BhupeshDeng, QiliangPsarras, SteliosDeNicola, GinaPsenicka, MartinDent, Joseph A.Psurski, MateuszDenvir, Martin A.Puglisi, RitaDergunov, SergeyPuig, BertaDeseure, KristofPulido, RafaelDeshmukh, RupeshPuniya, Bhanwar LalDeshpande, GauraviPuppo, FrancescaDeslouches, BerthonyPüschel, GerhardDesmet, TomPyatibratov, MichaelDeth, RichardPyka, AlinaDetmar, MichaelQi, FengDever, JaneQi, JunpengDeyev, Igor E.Qi, Phoebe X.Dhaenens, Claire-MarieQian, ChunqiDhankher, Om ParkashQiao, HuanyuDhar, DebanjanQimron, UdiDharmaraj, SelvakumarQin, Ren YiDi Bussolo, ValeriaQneibi, MohammadDi Dato, ValeriaQuan, TaihaoDi Filippo, MarziaQuattrocelli, MattiaDi Fiore, AnnaQuinlan, Roy A.Di Foggia, MicheleRabanal Anglada, FrancescDi Fonzo, AlessioRabbani, GulamDi Giuda, DanielaRaccuia, Salvatore AntoninoDi Liegro, ItaliaRadeghieri, AnnalisaDi Maro, AntimoRademaker, GillesDi Martino, Maria TeresaRader, ChristophDi Martino, OrsolaRadisavljevic, ZivDi Micco, SimoneRadko, LidiaDi Michele, AlessandroRadominska-Pandya, AnnaDi Rocco, GiulianaRadosavljević, TatjanaDi Sotto, AntonellaRadosinska, JanaDiaferia, CarloRadosinski, LukaszDias, JulianaRadulescu, CristianaDiaz, ArturoRafacho, AlexDíaz, CaridadRafiq H Siddiqui, MohammedDidangelos, TriantafyllosRagavan, MukundanDiepold, AndreasRagg, EnzioDietz, Allan B.Raggi, CarlaDijana Škorić, DijanaRagnini-Wilson, AntonellaDijkstra, Bauke W.Rahman, AzizurDikeakos, JimmyRaimondi, ClaudioDimasi, NazzarenoRaimondo, DomenicoDiminic, JankoRaimundo, NunoDimitroff, Charles J.Raina, SatishDing, YousongRajagopal, SudarshanDiomede, FrancescaRajakumar, NagalingamDiomede, LuisaRajarapu, Swapna PriyaDissel, StephaneRajput, AkankshaDittadi, RuggeroRajput, Vishnu D.Dittmar, GunnarRamachandiran, IyappanDittmer, JurgenRamachandran, AnupDivella, RosaRamalingam, NagendranDjabali, KarimaRamanadham, SasankaDjamgoz, MoustafaRamirez, Giuseppe A.Djedaïni-Pilard, FlorenceRamon Osman, TasanDjokovic, RadojicaRamos, Adrián M.Długaszewska, JolantaRampanelli, ElenaDługosz, EwaRando, DanielaDmitryjuk, MałgorzataRando, Maria MargheritaDmitrzak-Węglarz, MonikaRankin, GaryDo, ThuyRao, Gundu H.R.Dobrikova, Anelia G.Raouche, SanaDobryszycki, PiotrRappocciolo, GiovannaDocea, Anca OanaRashid, Mohammad HarunDocimo, TeresaRashkov, IliyaDoda, Sai ReddyRasinger, Josef DanielDolashka, PavlinaRaspor, MartinDoležal, KarelRatajewski, MarcinDoligalska, MariaRathinasabapathy, AnandharajanDolzhenko, Anton V.Rato, Ana ElisaDomingo-Almenara, XavierRätsep, MatthewDomingos, Pedro M.Rau, IleanaDomingues, PedroRavaioli, StefanoDomingues, SaraRavalli, SilviaDomitrović, RobertRavanidis, StylianosDonati, IvanRavera, EnricoDonato, RosarioRavid, TommerDong, ShenRawel, HarshadraiDong, YufengRay, SupriyoDonno, DarioRaymond, JosetteDontsova, Olga A.Reardon, PatrickDormoy, ValerianRébé, CédricDorotkiewicz-Jach, AgataRebelo, SandraDos Santos, CatarinaRebuffat, SylvieDos Santos, PatriciaRechkoblit, OlgaDos Santos, Reinaldo SousaRecinella, LuciaDosio, FrancoReddy, Sai T.Dosztanyi, ZsuzsannaReddy, SakamuriDouki, ThierryReffuveille, FanyDouma, MountasserReimann, Regina R.Doyen, AlainReinhardt, ChristophDoytchinova, IriniReinke, HansDräger, GeraldReipa, VytasDragoš, AnnaReis, AnaDreschers, StephanRejinold, SanojDreyfus, GeorgesRemelli, MaurizioDrin, GuillaumeRemenyik, JuditDrobizhev, MikhailRemmas, NikolaosDrolet, Barbara S.Ren, XiaoyuanDrori, RanRen, YanrongDrosten, MatthiasRenault, NicolasDrummond, HeatherRequena, DavidDruzbicki, KacperRequena, JesusDrużyńska, BeataResendiz, MarinoDu Plessis, Heinrich WResnick, AndrewDuarte, NoeliaRestani, LauraDubiel, WolfgangReszka, EdytaDubrez, LaurenceRetamal, MauricioDucaiova, Zuzana KovalikovaReverter, MiriDuchateau, NicolasReyes-Batlle, MaríaDudová, IvaReymond, Jean-LouisDuenas, MontserratReynoird, NicolasDuennwald, Martin LRezaei-Ghaleh, NasrollahDufour, AntoineRezvani, KhosrowDullaart, Robin Pieter FrankRhazi, LarbiDumas, PhilippeRhett, Joshua MatthewDumetz, FranckRho, Jung-RaeDunn, Michael F.Rhodes, Lyndsay V.Dupont, JoëlleRialland, MickaëlDurek, ThomasRiaz, AdnanDursun, SerdarRiazuddin, SaimaDutta, ArijitRibaudo, GiovanniDutta, PalashRibeiro Macedo, SandraDutta, RahulRibeiro, ApoenaDzhemileva, LilyaRibeiro, LauraDziendzikowska, KatarzynaRibot, JoanE Quelle, DawnRiccardi, ClaudiaEamens, Andrew L.Ricci, ClaudiaEbani, Valentina VirginiaRiccò, MatteoEbel, FrankRichards, Joanne S.Ebihara, LisaRichardson, BruceEbrahim, WeaamRichardson, LaurenEcheverria, FernandoRichter, MagdalenaEckhart, LeopoldRiddiford, Lynn M.Ediriweera, Meran KeshawaRieger-Christ, Kimberly MEduati, FedericaRifler, Jean-PierreEdwards, Dylan R.Riljak, VladimírEfimova, Evgeniya V.Rise, PatriziaEgea, JavierRispail, NicolasEggeling, LotharRissiek, BjörnEgger, BorisRittmann, SimonEhata, ShogoRivera, GuillermoEide, DavidRivera-Monroy, Zuly JennyEiring, AnnaRivera-Pérez, CrisalejandraEisel, Ulrich L. M.Rizzi Sanches, ElenEisenbach, MichaelRizzo, CarmenEjidike, Ikechukwu P.Rizzuti, BrunoEkielski, AdamRobaszkiewicz, AgnieszkaEkVitorin, JoseRobert, QuinnEl-Amri, ChahrazadeRobertson, JaniceElbayoumi, Tamer ARobinson, Stacey L.Elble, Randolph C.Robson, Matthew JamesEldeeb, MohamedRocha, Emily M.Eleftheriadis, TheodorosRocha, JoãoEleftheriou, CyrilRocha, Murilo R.Elfalleh, WalidRochais, ChristopheElizondo-Montemayor, LeticiaRockenfeller, PatrickEllgaard, LarsRodenbeck, Stacey DineenEllis, Lee D.Rodrigo-Comino, JesusEl-Maarouf-Bouteau, HayatRodrigues, Célia F.ElMallah, Mai K.Rodrigues, ElianaElosegui-Artola, AlbertoRodrigues, Joaquim RuiEmilsson, ValurRodrigues, MárcioEmmert, SteffenRodríguez González, VicenteEndres, KristinaRodriguez, AlvaroEngelberg, DavidRodríguez, Manuel S.Engelberth, JurgenRodriguez-Esteban, RaulEngelhardt, Paul FriedrichRodríguez-Pérez, Ana IsabelEngler, AnnaRodriguez-Vita, JuanEnos, Reilly T.Roeters, Steven JoopEnyedi, Kata NóraRogasevskaia, TatianaErdmann, Kai SvenRogers, KimEriani, GilbertRogers, SimonErickson, PeterRoh, SanghoErikson, KeithRoh, TaehyunErjavec, Gordana NedicRohde, John R.Eroglu, AbdulkerimRolla, SimonaErsilia, AlexaRolland, FilipErythropel, Hanno C.Rolle, LucaEsatbeyolgu, TubaRomanelli, Maria NovellaEspino, JavierRomano, PatriziaEsposito, FrancescoRomanov, GeorgiiEsquifino, Ana IsabelRománszki, LorándEsseltine, JessicaRoncevic, TomislavEsser, JenniferRong, Chan KuanEstevez, AliciaRosas Cárdenas, Flor De FátimaEstévez, JorgeRosca, Ana MariaEthirajan, AnithaRoseanu, AncaEulenburg, VolkerRosellò Catafau, JoanEvgen’ev, Mikhail BRosenberger, Thad A.Evidente, AntonioRosenberry, TerroneEyer, LuděkRosencrantz, Ruben R.Ezhov, MaratRosenzweig, DerekEzquerra-Brauer, Josafat-MarinaRosenzweig, Steven A.Fabiani, RobertoRoss, AvenaFabris, LindaRoss, JenFacelli, Julio CRoss, SamirFactor-Litvak, PamRossi, ElisaFaenza, IreneRossignol, JulienFaísca, PatríciaRossing, MariaFalanga, AnnaritaRosso, NataliaFalciani, ChiaraRossotti, Martin A.Fällman, MariaRostovtseva, TatianaFallowfield, JonathanRoszczenko-Jasinska, PaulaFang, ChengRoterman, IrenaFang, Evandro F.Roth, MichaelFang, QingmingRothbauer, MarioFang, XianjunRothbauer, UlrichFang, XingRottbauer, WolfgangFania, LucaRoucou, XavierFanizzi, Francesco PaoloRouhier, Matthew F.Fantini, ElioRousset, RaphaëlFarmaki, TheodoraRoy, AnuradhaFarmer, DianaRoy, Peter J.Farris, Alton BradRoy, Swapan KumarFarsky, Sandra H.p.Rozhon, WilfriedFato, RomanaRuben, PereiraFauconnier, Marie-LaureRubio, LourdesFaudry, EricRuckdeschel, KlausFavre-Réguillon, AlainRudack, TillFazakerley, DanielRüdiger, StefanFedorova, Olga V.Rudolph, JohannesFei, TongRuelland, EricFeintuch, AkivaRuf, Viktoria C.Feith, DavidRuffinatti, Federico AlessandroFeldman, RicardoRuhanya, VurayaiFeldo, MarcinRuiz Mendez, MariaFeliciello, IsidoroRuiz, MarioFÉLIX, RUTE CASTELORuiz-Canela, MiguelFeng, Hua-JunRuiz-Gómez, GloriaFentiman, IanRuiz-Hurtado, GemaFerguson, Matthew L.Ruiz-Moyano, SantiagoFernandes, João Paulo S.Ruml, TomasFernandes, Rosa Cristina SimoesRunge, Kurt W.Fernandes, Tiago G.Ruocco, NadiaFernández Ochoa, ÁlvaroRushworth, StuartFernández, María DoloresRusnati, MarcoFernández-Álvarez, AlfonsoRusso, MariaFernandez-Jimenez, NoraRusso, MarinaFernández-Moreno, MercedesRuvo, MenottiFernandez-Palomo, CristianRuysschaert, Jean MarieFernandez-Recio, JuanRuzov, AlexeyFernández-Rodríguez, JavierRuzza, ChiaraFernandez-Vizarra, ErikaRybakowska, IwonaFernig, DavidRybka, KrystynaFerraguti, FrancescoRychkov, GrigoriFerrao, PetranelRzymowska, JolantaFerrari, MarcoSaad, MohamedFerraroni, MartaSaba, JulieFerreira, ChristinaSabatier, Jean-MarcFerreira, HelenaSack, UlrichFerreira, Leonardo G.Saddala, Madhu SudhanaFerreira, MarceloSádecká, JanaFerreras, José M.SADOVSKAYA, IrinaFerreri, CarlaSaele, ØysteinFerrer-Montiel, AntonioSagheddu, ClaudiaFerri, Jose M.Saha, SajibFerri, MauraSaha, SubhasishFerro, Emer SuavinhoSaha, SuryaFich, Eric A.Saheki, TakeyoriFiester, SteveSahni, AbhaFigueroa-Espinoza, Maria CruzSahu, PranavFilek, MariaSaiardi, AdolfoFilgueiras, CamilaSaikumar, PothanaFilip, AdrianaSaiman, Mohd ZuwairiFilipeanu, CatalinSaini, Ramesh KumarFilipovic, MiodragSainlos, MatthieuFillmore, HelenSaisho, YoshifumiFinck, Brian N.Saito, KosukeFinol-Urdaneta, Rocio K.Saito, YoshinoriFiore, VincenzoSaitoh, KenjiFiori, MarianaSaitoh, MasaoFisar, ZdenekSakai, HiromiFischer, HenrikeSakai, HiromichiFischer, ReinhardSakamoto, KensakuFischer, UtzSala, RobertoFisher, JedSałaga, MaciejFisher, PaulSalata, CristianoFisk, John D.Salazar, IvanFiszer, AgnieszkaSalehi, Hassan S.Fitter, JörgSalehzadeh-Yazdi, AliFitzgerald, DavidSalerno, LoredanaFizeșan, IonelSaller, MaximilianFlorán, BenjamínSalman, MootazFlorek, EwaSalvador, ArmindoFlores, IgnacioSalvati, EricaFlórez-Fernández, NoeliaSalzano, AndreaFloris, MatteoSalzer, IsabellaFlorsheim, EstherSalzet, MichelFondufe-Mittendorf, Yvonne N.Samaja, MicheleFonin, AlexanderSamaniego, RafaelFONTANA, GIANFRANCOSamarakoon, RohanFontés, MichelSambuceti, GianmarioFord, LaurenŠamec, DunjaForest, FabienSamhan-Arias, AlejandroForini, Francesca S.Samiec, MarcinForlani, FabioSamson-Himmelstjerna, Georg VonFormenti, FedericoSamsonov, SergeyForte, ElenaSanchez De Groot, NataliaForte, MaurizioSánchez, Diego HernánFossa, PaolaSanchez, OttoFradin, ChantalSanchez-Quesada, José LuisFragoso, AlexSanchiz, ÁfricaFraietta, RenatoSandbichler, AdolfFranca, Eduardo FSanders, Alison P.Franca, Tanos Celmar CostaSanders, YanFrancelle, LaetitiaSandström, CorinneFranceschini, NicolaSanejouand, Yves-HenriFrancisco, TâniaSanfeliu, CoralFrancklyn, Christopher S.Sanguinetti, MaurizioFrancuzik, WojciechSanjeet, MehariyaFrank, Craig L.Sanjeewa, Kalu Kapuge AsankaFrank, SašaSantamaria, RitaFranke, JakobSantamaria, SalvatoreFranzoni, GiuliaSantana, Andrés GonzálezFratantonio, DeborahSantana-Codina, NaiaraFrau, JuanSantiago-Vázque, LoryFrecer, VladimirSantin, IzortzeFreeman, Michael F.Santini, AntonelloFretté, XavierSantonico, ElenaFrick, LucianaSantoni-Rugiu, EricFricker, RosemarySantos, Cleydson Breno R.Friedman, ThomasSantos, HugoFröhlich, EleonoreSantos, KlebsonFrugis, GiovannaSantos, Maria M. M.Fu, BeideSantos, Raquel De Cássia DosFu, PengSantoso, NettyFu, Shu-LingSantucci, AnnalisaFu, ZhengSantulli, GaetanoFujioka-Kobayashi, MasakoSapozhnikov, Alexander M.Fujita, MasakiSapudom, JiranuwatFujita, MikakoSarasan, ViswambharanFujita, WakakoSarasua, Jose-RamonFukuchi, SatoshiSarkar, AmritaFukuda, KenjiSarkar, AurijitFukuda, SeijiSarnataro, DanielaFukudome, AkihitoSartini, DavideFuliaş, Adriana VioletaSartorelli, PatriciaFumiaki, ItoSartorius, KurtFurneri, Pio MariaSasser, Jennifer M.Furse, SamuelSastre, LeandroFurukawa, KoichiSato, KousukeGabbia, DanielaSato, ShuzoGábor, ParagiSatoh, KatsuyaGadermaier, GabrieleSattler, WolfgangGaebel, RalfSauguet, LudovicGaedcke, JochenSavchenko, Tatyana V.Gaetani, LorenzoSazonova, Margarita A.Gagliano, NicolettaScadding, Glenis KathleenGagliano, TeresaScampicchio, MatteoGaidano, ValentinaScanlon, Karen M.Gaj, RenataScarpa, Emanuele-SalvatoreGál, PeterScarpa, MelaniaGala, Rikhav PScarselli, MarcoGalardi, SilviaSchaaper, RoelGaldiero, Maria RosariaScheggi, SimonaGaleazzi, RobertaSchelvis, JohannesGalindo, Máximo IboSchengrund, Cara-lynneGalkina, Svetlana I.Schenone, SilviaGall Troselj, KoraljkaScherf, Katharina A.Gallagher, Emily JaneSchiefer, Ana IrisGallant, NathanSchiemann, OlavGallegos Monterrosa, RamsesSchild-Poulter, CarolineGallegos-Arreola, Martha PatriciaSchindler, Charles W.Gallo, GianlucaSchjoldager, Katrine T.-B.G.Gallo, MonicaSchmid, DiethartGalluccio, Micheleschmidt, FlorianGalpert, DeborahSchmidt, KristinaGalvan, Daniel LSchmidtke, GunterGama, SofiaSchmitz, MatthiasGan, SamuelSchmitz, UlfGan, WenjianSchneider, DavidGangadaran, PrakashSchneider, Ian C.Gao, BeiSchneider, RobertGao, JuSchnell, RobertGao, ZheSchoenberg, Daniel R.García Fernández, EmilioSchoenhagen, PaulGarcía Gonzalo, FrancescSchohn, HervéGarcía Mateu, MauricioSchols, DominiqueGarcia, SamuelScholtz, VladimírGarcía-Angulo, PenélopeSchomacher, LarsGarcia-Bailo, BibianaSchön, JenniferGarcia-Borron, Jose CarlosSchonewille, MartijnGarcia-Bustos, Jose F.Schrum, AdamGarcía-Cañadilla, PatriciaSchubert, MarioGarcia-Gallego, SandraSchubert, StephanieGarcía-García, Rosa MaríaSchueler, JuliaGarcía-González, VíctorSchultz, David JGarcia-Jares, CarmenSchulze-Tanzil, Gundula GesineGarcía-Malinis, Ana JuliaSchwab, RebeccaGarcía-Ortega, LucíaSchwaminger, SebastianGarcia-Pardo, JavierSchwartz-Albiez, ReinhardGarcia-Vallve, SantiagoScicchitano, PietroGarner, Bianca L.Scoffone, Viola CamillaGarozzo, DomenicoScott, IanGarrett Da Costa, RafaelScotti, MarcusGarrido, SauloScozzafava, AndreaGarriga, PereScuruchi, MicheleGasic, IvanaSebastiani, FedericoGašić, Uroš M.Sebestik, JaroslavGáspári, ZoltánSecci, DanielaGaszner, BalázsSeco-Rovira, VicenteGatto, FrancescaSeeherunvong, TossapornGauld, James W.Seeliger, JessicaGaustad, Ann HelenSegura, Miguel FGautieri, AlfonsoSek, SlawomirGavara, NúriaSekino, YoheiGavín, RosalinaSelent, JanaGavira, JoséSelivanova, Svetlana V.Gaweł-Bęben, KatarzynaSemina, Ekaterina V.Gawlik-Dziki, UrszulaSenel, MehmetGawri, RahulSengupta, BidishaGe, WanzhongSeo, DaisukeGe, XinSepuru, Krishna MohanGeisler-Lee, JaneSequeda-Castañeda, Luis G.Gell, David A.Serefko, AnnaGeller, Herbert M.Sergeant, KjellGelpi, Josep LluisSergey, NetesovGemma, SandraSergi, ConsolatoGendaszewska-Darmach, EdytaSergi, DomenicoGenetos, Damian CSerino, GiovannaGenilloud, OlgaSerrano, Juan Alfonso AyalaGenisheva, ZlatinaSerrano, MaríaGeorgiades, SavvasSerrano-Pertierra, EstherGeorgiev, VasilSethi, GautamGerard, HeleneSeto, ToshiyukiGerdol, MarcoSetzer, William N.Gergs, UlrichSevastre, BogdanGermain, PierreSevilimedu Veeravalli, SathyanarayananGerman, Nadezhda A.Sevvana, MadhumatiGessner, GuidoSeydel, TiloGeszke-Moritz, MałgorzataSezgin, ErdincGhavami, SaeidSgambato, AlessandroGhayor, ChafikSgherri, CristinaGhigo, AlessandraShackelford, JuliaGhirga, FrancescaShackelford, Rodney E.Ghodake, GajananShakya, AkhileshGhosh, SanchitaShao, FangweiGhysels, AnShao, PengGiacomazza, DanielaSharma, AnketGiamperi, LauraSharma, Bhesh RajGiampietro, LetiziaSharma, IshaGiannoni, PatriziaSharma, Piyush SindhuGiannotti, Marina InésShaul, Yoav D.Giansanti, FrancescoShaw, Pang-ChuiGibbs, BernhardShaw, Robin M.Gil, Jesus PerezShcherbakova, DariaGiles, GregoryShcherban, Andrey B.Giles, WayneShchyogolev, SergeiGiliopoulos, DimitriosShe, Zhi-GangGillespie, James W.Shee, KevinGillet, GermainShellaiah, MuthaiahGillingham, Melanie B.Shen, HaihongGilmour, SusanShen, LongGimpel, MatthiasShen, QiangGinsberg, Stephen D.Shen, WeiningGiordano, AntonioShen, XiulongGiordano, SamanthaShen, YiGiorgio, SelmaShen, YulongGiovagnoli, RaffaelaSheng, YiGiovannelli, PiaShi, JunchaoGiovannoni, RobertoShi, LeiGiovarelli, MirellaShi, YanshuGiraffa, GiorgioShi, ZengqianGiraldo, JesúsShi, ZhiGirao, HenriqueShiao, Young-JiGiri, HemantShibata, HidekiGitlin-Domagalska, AgataShidoji, YoshihiroGiudetti, AnnaShieh, Tzong-MingGiuffrida, PaoloShifman, JuliaGiuliano, MichelaShigeta, YasuteruGjörloff Wingren, AnetteShih, Chun-KuangGladysheva, Inna P.Shih, Yin-HwaGlass, Karen C.Shikama, YosukeGlatt, SebastianShikov, AlexanderGlazer, LilahShim, HyunboGloess, Alexia N.Shimada, YasuhitoGlover, Kerney JebrellShimizu, KatsuhikoGlynn, StevenShimizu, TakahikoGnoni, AntonioShinohara, MinoruGodlewski, GrzegorzShin-Yi, MarzanoGodzien, JoannaShiomi, YasushiGoff, Wilfried LeShiraishi, FumitoGohda, EiichiShirakawa, TetsuoGoldberg, AlfredShirato, KenGoldeman, WaldemarShirole, NitinGoldsmith, ElizabethShiuchi, TetsuyaGolemi-Kotra, DasantilaShoshan-Barmatz, VardaGolenberg, Edward M.Shriver, LeahGolombick, TerryShugo, SuzukiGomer, RichardShukla, KirtikarGomes, AndreiaShukla, Sanjay K.Gomes, Bruno CostaShvets, ElenaGomes, Joao RSiegel, AmandaGomes, NelsonSieira, RodrigoGomes, PedroSieniawska, ElwiraGómez De Cedrón, MartaSikazwe, DonaldGomez-Gutierrez, JorgeŠikurová, LibušaGómez-Serranillos, M. PilarSilachev, DenisGominho, JorgeSileikyte, JustinaGonçalves, CatarinaSilman, IsraelGonçalves, LídiaSilva, Alexandre RGonda, SandorSilva, AndreGong, JiruiSilva, Susana N.Gong, RuiSilva-Cunha, ArmandoGong, Seung PyoSim, JaehoonGonzales, Eric B.Simanshu, Dhirendra KGonzalez Halphen, DiegoSimirgiotis, MarioGonzález Laredo, Rubén F.Simões, NelsonGonzález Maglio, Daniel H.Simon, IstvanGonzalez Rosa, Juan ManuelSimon, JorgeGonzález, Florenci V.Simon, LizGonzalez, ZoiloSimon, MichelGonzález-Díaz, HumbertSimone, AnfossiGonzález-Díaz, HumbertoSimone, Angela DeGonzalez-Fernandez, TomasŠimoník, OndřejGonzalez-Navarro, HerminiaSimonsson, StinaGonzález-Reimers, EmilioSinanoglou, VassiliaGonzalez-Stuart, Armando EnriqueŠindlerová, LenkaGooley, PaulSingh, AnandGophna, UriSingh, AnilGoreham, ReneeSingh, Brijesh KumarGoto, AkiteruSingh, SomnathGöttert, ThomasSingla, BhupeshGötz, Klaus-PeterSinharoy, PritamGoumans, Marie-JoséSiniscalco, DarioGovaere, OlivierŠinko, GoranGozal, EvelyneSintim, HermanGrabacka, MajaSiódmiak, JacekGrabarczyk, MałgorzataSipos, KatalinGrabsztunowicz, MagdaSiristatidis, CharalamposGraczyk-Jarzynka, AgnieszkaSissler, MarieGraether, SteffenSisto, MargheritaGraeve, LutzSitdikova, GuzelGraille, MarcSivaraj, Kishor KumarGrandi, GuidoSkała, EwaGrandjean, Jan G.Skandalis, SpyrosGranja, Pedro L.Skorko-Glonek, JoannaGranja, TiagoSkorska, AnnaGrant, MelissaSkowyra, DorotaGraslund, TorbjornSkrypnik, KatarzynaGrasso, GianvitoSkrzypek, KlaudiaGrativol, CliciaSkrzypski, MarekGrattan, Lynn M.Skuja, SandraGrayfer, LeonSkupin-Mrugalska, PaulinaGrayson, Dennis R.Skurikhin, Evgenii GermanovichGraziano, GuellaSkvortsov, DmitryGreening, DavidSlatter, M. A.Gregáň, JurajSleigh, AlisonGregory, Karen JSlim, SmaouiGreive, SandraŚliwińska-Wilczewska, SylwiaGribskov, MichaelSłomińska-Wojewódzka, MonikaGriffiths, WilliamSlominski, AndrzejGrigorenko, Elena L.Słomka, ArturGrimm, Marcus O. W.Słomko, JoannaGriñán-Ferré, ChristianSmalley, Joshua L.Grishin, Alexander V.Smeriglio, AntonellaGrocholewicz, KatarzynaŚmieszek, AgnieszkaGromadzinska, JolantaSmith, DavidGrones, PeterSmith, EricGroschner, KlausSmith, JeffreyGroveman, BradleySmith, JenniferGroves, MatthewSmith, Jeremy C.Gruber, ChristianSmith, Micholas DeanGrubić Kezele, TanjaSmith, Robert E.Grudnik, PrzemyslawSmits, Kim M.Grudzien-Nogalska, EwaSmolková, KatarinaGrunt, ThomasSnider, Ashley JGrusch, MichaelSnider, Mark J.Grzmil, PawełSnowden, TimothyGu, WenyiSoares, RaquelGude, NatalieSobeh, MansourGueguen, NaïgSobieszczuk-Nowicka, EwaGuevara González, RamónSobol, RobertGuharoy, MainakSobrado, PabloGuiard, BrunoSodi, Anna MensualiGuibert, ChristelleSodt, Alexander J.Guillén-Sánchez, Dominico ASoini, SannaGuix, Francesc XavierSoleimanpour, Arash S.Gulati, GauravSoler, Miguel ÁngelGüldiken, NurdanSolingapuram Sai, Kiran KumarGulei, DianaSoltész, AlexandraGullberg, DonaldSompol, PradoldejGullon, BeatrizSoncin, FrancescaGullon, BetatrizSong, KenanGunasekera, SunithiSong, Myoung-ChongGuo, XinSong, YuyuGuo, ZhengSoond, Surinder M.Gupta, RisheinSoós, VilmosGupta, SupritSophianopoulou, VickyGurrapu, SreeharshaSoran, Maria-LoredanaGurskaya, Nadya G.Sorensen, JohnGursky, JanSorescu, Ana AlexandraGushchin, IvanSorgen, Paul L.Gushina, IrinaSorrell, J. MichaelGussago, CristinaSorrenti, ValeriaGutiérrez, LauraSorsa, TimoGutowska, IzabelaSoslau, GeraldGuzman, FannySoto, RobertGyöngyösi, MariannSoufan, OthmanGyurcsik, BelaSoulimane, TewfikHa, Jung-HeunSoundararajan, PrabhakaranHaas, NikolausSousa, Maria JoãoHadži, SanSousa, Sérgio F.Hafizi, SinaSouth, Paul F.Hagelueken, GregorSovadinova, IvaHagihara, MasakiSoveral, GraçaHahm, EunsilSowa, YoshiyukiHaitin, YoniSoyfoo, MuhammadHakkinen, LariSpagnol, GaëlleHála, MichalSpeijer, DaveHalaban, RuthSpella, MagdaHalder, Amit KumarSpence, RowenaHaley, John D.Spencer, Gaynor E.Hall, David H.Spies, MariaHallett, Maurice B.Spittau, BjörnHallmann, EwelinaSplichal, IgorHallsworth, J.E.Spratt, Donald E.Halmosi, RóbertSpray, DavidHamamoto, RyujiSpur, Bernd W.Hamblin, MichaelŚrednicka-Tober, DominikaHamdani, NazhaSridhar, JayalakshmiHamden, KhaledSrivastava, PranayHammers, Christoph M.Srivastava, SameerHammond, GerrySrivastava, SaurabhHamson, DwayneStaal, JensHan, XiaobinStadlbauer, PetrHanafy, Nemany A. N.Staiger, DorotheeHanania, MichelStana, Anca - DanielaHandelsman, DavidStanek, AgataHannun, YusufStanford, StephanieHano, ChristopheStange, KatjaHanschen, FranziskaStanisz, BeataHansíková, HanaStankovic, MilanHanski, LeenaStanley, Jone A.Hanson, JulienStanley, RobinHao, JiaqingStano, PasqualeHao, MingangStarek, MałgorzataHao, NanjingStatti, GiacarloHappel, Christine M.Stear, MichaelHara, AkiraStefanou, StefanosHardman-Smart, JonathanStefanowicz, JoannaHardtke-Wolenski, MatthiasSteger, GerhardHarijan, Rajesh KSteinbeck, ChristophHarm, StephanSteiner, JenniferHaro, DiegoSteiner, Joseph P.Harper, JamesStellato, FrancescoHarpsøe, KasperStenbeck, GudrunHarris, DanniStepanenko, Olesya V.Harrison, FreyaStepanova, Anna A.Harrison, Paul H. M.Stephens, GaryHarrop, RichardStephens, Jacqueline M.Hartman, MariuszSterckx, YannHartman, Matthew C.T.Steri, MaristellaHarvey, PetaSterling, James D.Hasanzadeh Kafshgari, MortezaSterling, JulieHaseltine, CynthiaSterner, OlovHashemolhosseini, SaidSteuer, ChristianHashimi, HassanStewart-Ornstein, JacobHashimoto, MakotoSticht, HeinrichHashimoto, MasaruStilhano, Roberta S.Hassell Jr., James E.Stimamiglio, Marco AugustoHastings, Philip J.Stines-Chaumeil, ClaireHatanaka, TomomiStinghen, Andréa Emilia MarquesHatano, AkihikoStoean, CatalinHatano, TakuStojakowska, AnnaHattori, MitsuharuStojanov, MilosHaubruck, PatrickStojko, JerzyHaupt, Larisa M.Stojko, RafałHawinkels, LukasStone, Kari L.Hawkins, MichelleStone, Trevor W.Hawrył, Anna M.Stone, William L.Hayashi, Mariko KatoStoppelkamp, SandraHayashi, MikioStork, Christian J.Hayashi, Mirian Akemi FuruieStorr, TimHayen, HeikoStoyanova, AlbenaHe, Ming FangStradner, Martin HelmutHeath, GeorgeStraight, PaulHeaton, Steven M.Strappazzon, FlavieHećimović, Silva KatuŝićSträter, NorbertHeddleston, JohnStrati, Irini F.Hedfalk, KristinaStraus, Suzana K.Heiker, John TStravopodis, Dimitrios J.Heinbockel, ThomasStrehmel, VeronikaHeine, ThomasStrelnikov, Vladimir V.Héja, LászlóStruzik, JustynaHelbert, WilliamStrzelecki, DominikHendrickson, TamaraStuart, David T.Henen, MorkosSu, JingHengesbach, MartinSu, JiyongHenkel, JaninSu, Mei-TszHeras Benito, ManuelSu, ZhaoqianHeras, FranciscoSu, Zheng-YuanHerde, MarcoSuarato, GiuliaHeredia-Guerrero, José AlejandroSuciu, Bogdan AndreiHering, ThomasSugawara, AkihiroHermenean, AncaSugaya, MakotoHermida Prieto, ManuelSugii, HidekiHernández, Eliud AvierSugimoto, KeiichiroHernandez, Jean-FrançoisSugimoto, MasahiroHernandez-Alonso, PabloSuh, Hyung JooHernández-Cifre, José GinésSuleria, Hafiz Ansar RasulHernandez-Olmos, VictorSullivan, John StephenHéroux, PaulSumara, GrzegorzHerrera, FedericoSumbayev, VadimHertzler, Philip LSun, ChaominHerzig, VolkerSun, GuoxingHetényi, CsabaSun, LichunHetherington, SandySun, WeiHettiaratchi, MarianSun, ZhixiongHeuertz, Rita M.Sun, ZhonghuaHeyduk, TomaszSunayama, HirobumiHeyer, Wolf-DietrichSundaresan, AlameluHibbitts, AlanSundberg, Eric J.Hiebert, PaulSuñer, Damian HeineHieronymus, ThomasSuomela, Jukka-PekkaHiggins, KhadineSuperti, FabianaHilhorst, MarcSur, SubhayanHinney, BarbaraSurguchov, AndreiHinton, DeborahSushchik, Nadezhda N.Hirasawa, NoriyasuSuwinska, KingaHiroaki, HidekazuSuzui, MasumiHirohata, SatoshiSuzuki, AyakoHirota, KiichiSuzuki, KeikoHirotsu, NaokiSuzuki, ShinyaHissa, BarbaraSuzuki, ToruHitchings, MatthewSvahn, OlaHjorth, MaritSvendsen, John S.Hlavaty, JurajSvetashev, VasilyHluska, TomášSwairjo, Manal A.Ho, Eric S.Swarbrick, CrystallHo, VincentSweeney, ElizabethHocart, CharlesŚwiderek, KatarzynaHochstrasser, MarkSwietnicki, WieslawHodgson, David R. W.Swiezewska, EwaHoebeke, JohanSykłowska-Baranek, KatarzynaHoelzl, FranzSýkora, JanHofer, AndersSynnott, NaoiseHoff, PaulaSyrovets, TatianaHoffman, Brad G.Sysolyatina, ElenaHoffman, Charles S.Szakiel, AnnaHoffmann, Lucia VieiraSzaleniec, MaciejHofmann, KaySzatmari, Erzsebet MariaHohenegger, MartinSzatmári, TündeHohmann, Miriam S. N.Szavits-Nossan, JurajHoldener, Bernadette C.Szczepanek, JoannaHoleček, MilanSzczepanik, Antoni MHolefors, AnnaSzczepankiewicz, AleksandraHolien, JessicaSzeberény, JózsefHolien, TorilSzegletes, ZsoltHolland, OliviaSzekalska, MartaHoller, EggehardSzeliga, JacekHolmes, NeilSzentmary, NoraHolt, Christine E.Szolnoky, GyozoHolzhauser, ThomasSzperlik, JakubHomma, KeiichiSzuts, DavidHondermarck, HubertSzweda, PiotrHonek, JohnSzweda, RozaHong, Jeong HeeSzymańki, PawełHong, YanSzymczyk, AgnieszkaHooda, JagmohanTachezy, JanHoover, Timothy R.Tae, Han-ShenHorakova, JanaTafelska-Kaczmarek, AgnieszkaHori, MikaTafuri, SimonaHorisawa, SakaeTagliavia, MarcelloHorman, SandrineTagliazucchi, DavideHornedo-Ortega, RuthTaguchi, YoshihiroHorowitz, MiaTaha, MuhammadHorvath, DragosTahara, HidetoshiHorwich, Arthur L.Tahirov, Tahir H.Horwitz, BenjaminTailhades, JulienHorzmann, KatharineTaillandier, DanielHoshino, MasaruTajiri, KazutoHosoyamada, MakotoTakahashi, Reisuke H.Hosui, AtsushiTakahashi, ToshioHour, Tzyh-ChyuanTakaki, ManabuHoury, Walid A.Takano, KatsuraHouston, Kevin D.Takasawa, ShinHowell, BrianTakeda, SenHrenar, TomicaTakeuchi, HideyukiHsia, Shih-MinTaki, MasumiHsiao, Po-JenTalavera, MiguelHsieh, Feng-ChiaTalbot, Jeffery N.Hsieh, Tusty-JuanTalhinhas, PedroHsieh, YvesTalukdar, SarmisthaHsu, Pang-HungTaly, AntoineHu, Chang-minTam, James P.Hu, ChenlinTamasi, GabriellaHuang, Dorothy YuTambaro, SimoneHuang, Genin GaryTambe, MitaliHuang, HaoTamkovich, SvetlanaHuang, Hui-ChiTan, Du-XianHuang, JijunTan, Yaw SingHuang, JinTanabe, ShihoriHuang, Jui-YenTanaka, JunichiHuang, Kuang-TzuTanaka, NaokiHuang, MingTanaka, NobutadaHuang, MingQingTanaka, TakujiHuang, Shih-YiTanase, CorneliuHuang, StanleyTanase, CristianaHuang, Tze-SingTanc, MuhammetHuang, Wei-ChienTang, AihuaHuang, XudongTang, Bor LuenHuang, Yu-ChuenTang, JingHuda, NazmulTang, RenjieHudson, JamesTang, Soon YewHuerta, MiguelTang, XiangruHueso, MiguelTani, AkioHuet, SebastienTanyi, Janos L.Hugelshofer, MichaelTao, PengHull, J.JoeTao, YongshengHumphreys, DavidTarafder, SolaimanHumphries, BrockTarasov, AndreiHung, Chin-ChuanTarn, Woan-YuhHunt, PamelaTashima, KimihitoHuq, Md. AmdadulTashima, ToshihikoHur, Su-MiTata, Ada MariaHurlburt, Barry K.Tate, Shin-IchiHurtado-Guerrero, RamonTatenhorst, LarsHusnjak, KoraljkaTatsuta, TakashiHussein, AhmedTatulian, Suren A.Hutchins, Andrew PTatullo, MarcoHutmacher, DietmarTaupitz, MatthiasHwang, Jae SungTavanti, FrancescoHwang, Ming-JingTavares, JoanaHwangbo, CheolTavolari, SimonaHyink, Deborah PinsonTawara, KenHyldegaard, OleTaylor, Eric B.Hynes, Michael F.Tchetina, Elena V.Hyun, Tae KyungTchoghandjian-Auphan, AurélieHyvönen, MarkoTchórzewski, MarekI. Kalinin, VladimirTedeschi, ValentinaIaccino, EnricoTeisseyre, AndrzejIannotti, Fabio ArturoTeixeira, Ana LuísaIanora, AdriannaTeixeira, Fábio G.Iasevoli, FeliceTeixeira, MiguelIbrahim, AhmedTeklić, TihanaIconomidou, VassilikiTéllez-Téllez, MauraIdili, AndreaTellier, MichaelIdris, AdiTemeyer, Kevin B.Ientile, RiccardoTemussi, Piero A.Ierardi, EnzoTeng, YongIgarashi, KazueiTerao, MinekoIgual, MartaTerashima, HiroyukiIhmels, HeikoTerme, NolwennIida, KeiTerrinoni, AlessandroIkeh, MélanieTerryn, ChristineIlari, AndreaTerzopoulou, ZoeIliakis, GeorgeTeske, AndreasIlies, Marc A.Tesmer, JohnImada, KatsumiThan, Nandor GaborImai, KenichiThanabalu, ThirumaranImai, ToshioThet, NaingImamoto, YasushiThevenod, FrankImbimbo, BrunoThiel, KristinaImperatore, ConcettaThomas, WayneInaba, HiroshiThomes, PaulIndiveri, CesareThompson, DamienInga, AlbertoThoms, SvenIngle, ShaktiThor, StefanInoue, AtsukoThorpe, StephenInserte, JavierThounaojam, Menaka ChanuInta, DragosThummel, RyanInui, MasayukiThurnher, MartinInuzuka, HiroyukiTian, ShengIppoliti, RodolfoTian, Ya-ChungIqbal, SumaiyaTian, YuanIram, SurtajTicconi, CarloIranzo, OlgaTiera, Marcio JoséIrbäck, AndersTikhonova, IrinaIrmisch, SandraTimoshenko, Alexander V.Isaac, Vera Lucia BorgesTimson, David J.Isaacs, MarkTinti, AnnaIshibashi, NobuyukiTisné, CarineIshida-Yamamoto, AkemiTiso, NatasciaIshii, IsaoTixeira, RochelleIshii, TetsuroTkac, JanIshizaki, YasukiTocco, GraziellaIshmael, Jane E.Toda, TomohisaIskierko, ZofiaTodaka, DaisukeIskra, MariaTodea, AnamariaIsola, GaetanoTogashi, YuichiIsom, LoriToiu, AncaItabashi, YutakaToma-Fukai, SachikoIto, KazuoTomar, DhanendraIto, YujiTomaszewska, EmiliaIvanov, AndreyTomatsu, ShunjiIvanova, Juliana M.Tomczykowa, MonikaIwamoto, MasahiroTomi, FélixIwamoto, NaokiTomikawa, ChieIwaniec, UrszulaTomko, Robert J.Iwasaki, KengoToms, DerekIwasaki, TakashiTonello, FiorellaIwata, HisatakaTong, HongningIwata-Reuyl, DirkTong, JunchaoIyer, ShilpaTong, XinmingIzawa, Kazuhiro P.Tong, YaojunIzawa, TetsuyaTopin, JeremieIzzo, LuanaToppo, StefanoJachimska, BarbaraTordai, AttilaJacobs, HuguesTorres Vaamonde, José EnriqueJacobsen, Kristian MarkTorres, Adrian GabrielJager, Martine JTorres-Lagares, DanielJaguva Vasudevan, Ananda AyyappanTorres-Ramírez, EduardoJain, AnkurToto, AngeloJain, AnshikaTotoson, PerleJakas, AndrejaToubanaki, DimitraJakob, Roman P.Tõugu, VelloJakse, JernejToy, RandallJakubík, JanTrackman, Philip C.Jakubowski, WitoldTrader, DarciJames, Nicholas G.Traiffort, ÉlisabethJanda, ElzbietaTrakselis, Michael A.Janda, TiborTran, Anh NhatJangampalli Adi, PradeepkiranTratnik, Janja SnojJankovic, BrankicaTraw, BrianJantas, DanutaTrček, JanjaJappe, UtaTreebak, JonasJaquet, KorneliaTreinin, MilletJaramillo-Flores, MariaTresguerres, JesusJarosz-Wilkołazka, AnnaTribulova, NarcisJärvinen, Tero A. H.Trincone, AntonioJasiulionis, Miriam G.Tripathi, Jitendra KumarJaumot, JoaquimTríska, JanJayaraman, ShobiniTrivedi, DarshanJaźwińska, AnnaTronchere, HeleneJee, ShiouhwaTrouvelot, SophieJelińska, AnnaTrubiani, OrianaJena, Prasant KumarTrudel, DominiqueJeng, AnnaTruman, James W.Jennane, RachidTrusheva, BoryanaJensen, IngvillTruzzi, CristinaJeon, OjuTruzzi, Daniela R.Jeon, SookyoungTsafantakis, NikolaosJeon, Young HoTsai, Ang-ChenJeong, Byeong-ryoolTsai, Kun-LinJeong, Byung-HoonTsai, Pei-ShiueJia, ZongchaoTsai, Pei-YinJian, XingTsai, Shu-YaoJiang, ChengTsapogas, PanagiotisJiang, DazhiTsarouhas, KonstantinosJiang, Qiu-XingTse Seng, ChuahJiang, TaoTseng, Chih-HuaJiang, WeiTsermpini, Evangelia EiriniJiao, XuanmaoTsoi, HoJibran, RubinaTsotinis, AndrewJimenez, CarlosTsouh Fokou, Patrick ValereJiménez, CarlosTsuchiya, YuichiJimenez, Patricia TTsuda, HiroyukiJin, Jun-OTsuda, SakaeJin, LingTsugawa, HitoshiJin, TakashiTsuge, TomohikoJin, YuefeiTsujita, MakiJiri, VrbaTsukahara, ToshifumiJo, HyunilTsumuraya, TakeshiJohansson, Mats W.Tsuruta, DaisukeJohler, SophiaTuccinardi, TizianoJohn, RohanTucker, JulieJohnson, EricTucker, NathanJohnson, IanTudela, JoseJohnson, PaulineTufarelli, VincenzoJohnson, R. JeremyTuleja, MonikaJohnson, Shelby L.Tung, Tao-HsinJohnstone, DanielTupally, Karnaker R.Johnstone, ElizabethTupler, RossellaJohnstone, ScottTurano, PaolaJonas, StefanieTurk, JohnJones, George H.Turner, MarkJones, HuwTuukkanen, JuhaJones, Ian M.Tylova, EditaJones, James F. X.Tyson-Capper, AlisonJorge, J. C. LuisTytgat, JanJorrin-Novo, Jesus VTzen, Jason T.José Tauler Riera, PedroTzortzakis, NikosJose, Pedro A.Tzovenis, IoannisJoseph, BeneshUchida, LéoJoshi, Pushpa RajUchiyama, TaketoJourno, ChloéUhrig, R. GlenJozefczak, MarijkeUldrich, AdamJóźwiak-Bębenista, MartaUldrijan, StjepanJu, TingtingUlicna, LiviaJuarez, KarlaUllah, IrfanJubeau, SébastienUllah, MujibJulien, GronnierUmar, ShahidJung, Jee-hyungUngaro, FedericaJung, Ji HoonUnno, HideakiJurcek, OndrejUnoki, TakamitsuJuszczak, LesławUpham, Brad L.K. Karabagias, IoannisUrban, MilanKabelác, MartinUrban, PhilippeKabil, OmerUrbanc, BrigitaKabisch, StefanUrbanelli, LorenaKacaniova, MiroslavaUrbaniak, AlicjaKačániová, MiroslavaUrbanowicz, MarcinKaczanowski, SzymonUrbanska, Edyta MariaKaczor, Jan JacekUrra, Félix A.Kaczorowski, TadeuszUsuki, YoshinosukeKaddes, AmineUversky, VladimirKadlubowski, SlawomirUygun, SahraKafarski, PawełUzbekova, SvetlanaKafel, AlinaVaamonde, CarlosKagawa, NatsukoVaidotas, StankevičiusKai, LeiVaidyanathan, VenkateshKajtár, BélaVakonakis, JohnKalesh, KarunakaranValdés López, OswaldoKaliňáková, BarboraValenti, MariaKaller, MarkusValentim, AnaKalogiouri, NatasaValentine, Rudy J.Kalueff, AllanValenzuela, Juan LuisKamal, Fadia AliValis, MartinKamari, YehudaVallera, DanielKamei, YasutomiValsami, GeorgiaKaminski, KamilVan Aerschot, ArthurKamiya, MitsunobuVan Delft, Floris L.Kamzolova, SvetlanaVan Den Ende, WimKanakkanthara, ArunVan Der Nest, Magriet AKanata, EiriniVan Der Vegt, BertKanda, NaokoVan Doorn, RemcoKandasamy, Richard KumaranVan Helden, DirkKang, Byung-jaeVan Hellemond, Jaap J.Kang, CongBaoVan Houten, JudithKang, Hee-GyooVan Lith, SanneKang, HunseungVan Raaij, MarkKang, KyuhoVan Rossum, Huub H.Kang, SeungwooVan Waardenburg, RobertKang, Tae-HongVan Haute, LindseyKang, Young-HeeVance, DavidKano, HideakiVanderlei, Maria De FátimaKaplan, BarbaraVandooren, JenniferKarcz, DariuszVangala, Janakiram R.Karczmarzyk, ZbigniewVanharanta, SakariKardos, JuliannaVanin, Anatoly F.Kari, LilaVaquero, JavierKarnati, Konda ReddyVaradarajan, ShankarKarpinski, Tomasz M.Váradi, CsabaKarunakaran, SuneeshVáradi, GyörgyiKashiwagi, ShinichiroVarga, Zoltan M.Kaškonienė, VilmaVargas Jentzsch, PaulKasuga, KensakuVargas-Murga, LilianaKatada, KazuhiroVarikuti, SanjayKataoka, NaoyukiVaseva, Irina I.Kato, TakayukiVasic, VesnaKatsiougiannis, StergiosVassallo, NevilleKatsyuba, ElenaVassão, Daniel GiddingsKawahara, MasahiroVasylieva, NataliaKawashima, HidekiVatrella, AlessandroKawauchi, KeikoVaz, Daniela C.Kawczak, PiotrVazquez, AlexeiKawiak, AnnaVazquez, Elba S.Kayisli, UmitVeeramani, SureshKe, HengmingVega, Juan FranciscoKe, Liang-YinVégh, Attila GergelyKędziora, AnnaVeit, MichaelKeefover-Ring, KenVeldkamp, ChristopherKeinänen, MarjaVelgosova, OksanaKejík, ZdeněkVellecco, ValentinaKeller, LanceVellido, AlfredoKellner, StefanieVenditto, VincentKelly, Gregory M.Venkatesan, Jagadeesh KKelly, VincentVenkateswaran, SureshKelts, JessicaVera Ávila, Héctor RaymundoKemp, Michael G.Verde, FedericoKenney, Maria CristinaVerdeguer, FranciscoKenny, Nathan JamesVergara, DanieleKerscher, OliverVerma, RajniKetkar, AmitVerma, RanjitKezic, SanjaVerma, RatiKhalifa, ShadenVermaas, Joshua V.Khalil-Ur-Rehman, MuhammadVerna, RobertoKhan, HamidullahVerreault, MaïtéKhan, IrfanVersacci, PaoloKhan, NaeemVerspoor, KarinKhan, SajidVerzola, DanielaKhan, TanveerVeschi, VeronicaKhananshvili, DanielVetere, AmedeoKhashayar, PatriciaVetrano, StefaniaKhathi, AndileVibhute, Sandip M.Khatib, FirasVicario-Abejón, CarlosKhatib, SolimanVicens, Quentin JeromeKhazaei, HamidVicente, LauraKhedr, Mohammed A.Vicente-Manzanares, MiguelKhobta, AndriyVictor, BrunoKhodarahmi, RezaVictor, VictorKholmukhamedov, AndalebVictoria - Clos, M.Kholová, IvanaViegas, Sandra C.Khung, Yit LungVieira, AlexandreKhurana, NamrataViennet, ThibaultKieber-Emmons, ThomasVigetti, DavideKieffer, NicolasVíglaský, ViktorKieliszek, MarekVignini, AriannaKierzek, RyszardVignoli, AlessiaKiezun, MartaVigodner, MargaritaKikuchi, HaruhisaViguera, Ana RosaKikuchi, KiyoshiVijgenboom, ErikKim, BongleeViktorova, JitkaKim, Byung-SooVilela, AliceKim, Chang-EopVilela, Janice Miranda VasconcellosKim, Chu-YoungVillalobo, AntonioKim, DohoonVillar-Piqué, AnnaKim, Eun-HeeVillegas-Sepulveda, NicolasKim, Ha HyungVimalachandran, D.Kim, Hak JunVimberg, VladimirKim, HangunViña, DoloresKim, HoonVincent, DelphineKim, Hwan KeunVinet, RaúlKim, Hye KyongVingolo, Enzo MariaKim, InKyeomVinha, AnaKim, Jeong BeomVinjamur, DivyaKim, Jeung GonViola, IvanaKim, Ji-HunVirag, Jitka A.I.Kim, Jin-KyungVirgili, FabioKim, Jong-SangVishe, MaheshKim, Jung-HwanVitetta, LuisKim, JunilVito, PasqualeKim, Ki-HongVitoratos, EvangelosKim, KiyoungVitrac, HeidiKim, Kyeong-ManVives, JoaquimKim, KyoungtaeVives-Peris, VicenteKim, Kyung JinVizovišek, MatejKim, Mi-hyunVlachou, MarilenaKim, Min-SikVlková, EvaKim, Seung WookVoběrková, StanislavaKim, SoochongVogel, AlexanderKim, T. DoohunVögele, MartinKim, YongsooVogt, MartinKim, Yoon KiVolkov, VadimKim, Young JunVondracek, JanKim, Yun KyungVorontsov, DmitryKimbaris, AthanasiosVoss, MatthiasKimura, HaruhideVozella, ValentinaKimura, HidetoVulturar, RomanaKimura, SeisukeWada, JunKimura, TakefumiWadhwa, NavishKimura, TsuyoshiWagner, Kay DietrichKing, Benjamin L.Wagner, RenaudKinghorn, Kerri J.Wahli, WalterKinkorová, JuditaWahlsten, MattiKirberger, MichaelWakao, MasahiroKirby, Elizabeth D.Wako, HiroshiKirchmaier, AnnWałajtys-Rode, ElżbietaKirchmair, JohannesWaldum, HelgeKirimura, KohtaroWalker, Anthony L.Kirpekar, FinnWalker, Douglas G.Kirsch, ThorstenWalker, Mary K.Kiss, RobertWalker, Robert J.Kita, ToshihiroWall Medrano, AbrahamKitagawa, KyokoWallace, Heather M.Kitchen, PhilipWalsh, Laurence JKitsiouli, EIRINIWalter, JeskeKitzmüller, ClaudiaWan, LeiKizaka-Kondoh, ShinaeWan, ShibiaoKjaergaard, MagnusWan, ZhongxiaoKlatte-Schulz, FrankaWanders, Ronald J.A.Klein, Andrés D.Wang, Be-JenKlein-Júnior, Luiz CarlosWang, BinKlimontov, VadimWang, ChaoKlochkov, S. G.Wang, ChengKlocko, Amy L.Wang, Chin-KunKloczkowski, AndrzejWang, ChunKlöhn, PeterWang, Di-YanKluckova, KatarinaWang, GuliangKnaus, Ulla G.Wang, HuaishanKnez, JureWang, HuaweiKnoche, MoritzWang, Jee-ChingKnutsen, ErikWang, JianjunKober, KordWang, JiminKocerha, JannetWang, Jin’AnKocica, Mladen J.Wang, NanKociok, NorbertWang, NingKodali, MaheedharWang, RuiyongKodidela, SunithaWang, SaiKoelmel, JeremyWang, TianfangKoenen, RoryWang, WenqiKoeppe, Roger E.Wang, XiuminKoga, FumitakaWang, XuKogut, Michael H.Wang, YanKoh, Cho YeowWang, Yeng-TsengKohout, SusyWang, YifeiKoike, HarukiWang, YongqiangKojima, SeijiWang, ZhenKokoska, LadislavWang, ZhiqiangKokotos, GeorgeWard, RobertKoksharova, Olga A.Ward, Robert M.Kolář, MichalWärmländer, Sebastian K.t.s.Kolch, WalterWarzych-Plejer, EwelinaKolesińka, BeataWasan, EllenKolinski, AndrzejWashino, SatoshiKoltai, HinanitWatanabe, YoichiroKomar, AntonWautier, Jean-LucKomosinska-Vassev, KatarzynaWaxman, JoshuaKonc, JanezWAYE, Mary M.Y.Kondakova, IrinaWebb, R. ClintonKong Thoo Lin, PaulWebba Da Silva, MateusKong, JimingWeber, Daniel N.König, GerhardWebster, Keith AKonopelski, PiotrWei, KongchangKonstantinidis, Theocharis G.Wei, ShuoKonstantinov, Spiro M.Weidenhofer, JudithKonto-Ghiorghi, YoanWeinhold, AlexanderKopečny, DavidWeinzierl, RobertKopsahelis, NikolaosWeiser, Brian P.Korac, AleksandraWeitzman, MatthewKorányi, TamásWelch, AilsaKorczeniewska, OlgaWeldon, JohnKorfiatis, NikolaosWeldy, Chad S.Korol, SergiyWelling, MickKosakowska, OlgaWells, LauraKosova, KlaraWeltin, AndreasKostareva, AnnaWemmer, DavidKőszegi, TamásWen, Yu-ChingKothapalli, Kumar SDWen, ZhiweiKotloski, RobertWenda-Piesik, AnnaKottakota, ChandrasekharWeng, Mao-LunKottom, Theodore J.Westh, PeterKoufaki, MariaWeston, Leslie A.Koulen, PeterWeydt, PatrickKoupenova, MilkaWhatmore, JacquelineKourkoutas, IoannisWhelan, DerekKouroupis, DimitriosWhite, CarlKoussis, KonstantinosWhite, RobertKoutelidakis, Antonios E.Whitehead, Shawn N.Koval, MichaelWhitfield, Phil.Kovalenko, AndriyWicky Collaud, ChantalKovermann, MichaelWie, Myung-BokKovoor, AbrahamWieckowska, AnnaKoyama, NobuhiroWieczorek, EdytaKoyama, TomotsuguWiedemann, NilsKozakiewicz, AnnaWiener, ReuvenKoziorowska-Gilun, MagdalenaWiese, SebastianKozlakidis, ZisisWiesman, ZeevKozlova, ElenaWiesner-Reinhold, MelanieKraeuter, Ann KatrinWięsyk, AnetaKrag, Thomas OWightman, Emma L.Krammer, Eva-MariaWiglusz, KatarzynaKrasheninina, Olga A.Wijnveld, MichielKrashias, GeorgeWildemann, BrittKrásný, LiborWilkinson, Kevin AKrause, John RWilkinson, Miles F.Krautkramer, Kimberly A.Wilkowska, AgnieszkaKrawczyk, KonradWillemse, ElineKretz, MarkusWilliams, DanielKrga, IrenaWilliams, FrederickKristian, TiborWilliams, JesseKrokidis, MariosWilliams, MarshallKrumova, SashkaWilliams, MichelleKruszynski, RafalWilliamson, Ethel DianeKrzepiłko, AnnaWilson, Jeremy SomersKrzyściak, WirginiaWilson, PeterKrzystanek, MarekWiltschi, BirgitKu, Seung-YupWimmer, MonikaKuan, Yu-HsiangWimmer, ZdenekKuban-Jankowska, AlicjaWindshügel, BjörnKubatka, PeterWinkle, MelanieKubíková, ĽubicaWińska, KatarzynaKubinova, SarkaWirz, Richard E.Kubo, TaiWitte, AngelaKubo, TakanoriWittig, IlkaKubori, TomokoWlaź, PiotrKuča, KamilWnorowski, ArturKucharska, KarolinaWnuk, MaciejKucheryavykh, Lilia Y.Wnuk, StanislawKudryashova, Elena V.Woerman, AmandaKudryavtsev, Denis S.Wójciak, MagdalenaKufareva, IrinaWojtunik-Kulesza, Karolina AnnaKufel, JoannaWolf, PhillippKujawska, MałgorzataWołkow, PawełKuksa, Pavel P.Wong, Chi-MingKukula-Koch, WirginiaWong, Ching-OnKulbacka, JulitaWong, MauriceKulkarni, ShashankWong, Yung-SingKumar Mishra, VivekWong, YvetteKumar, AkhileshWood, David W.Kumar, DhirendraWoolf, Eric J.Kumar, GokhleshWorch, RemigiuszKumar, NarenderWorman, HowardKumar, PradeepWorsnop, Christopher JKumar, RajeevWorthmann, AnnaKumar, UjendraWoscholski, RudigerKumar, VijayWöstemeyer, JohannesKume, KensukeWozniak-Knopp, GordanaKunjukunju, SangeethaWree, AndreasKunoh, TatsukiWright, Gerard D.Kunsági-Máté, SándorWróbel, MariaKuo, Jen-MinWu, (Jason) BoyangKuo, Ming-TseWu, CenKurakula, KondababuWu, Chung-HsinKuramata, MasatoWu, Chung-YiKurgan, LukaszWu, ErxiKurien, Biji T.Wu, JenniferKurtenbach, StefanWu, Jin-MingKusaczuk, MagdalenaWu, Li-chenKusamori, KosukeWu, Long-feiKushiro, TetsuoWu, MengKusters, IljaWu, Ming-JiuanKusumanchi, PraveenWu, Min-HuanKusunose, KenyaWu, Ping-HsunKuter, KatarzynaWu, YingnianKutikhin, Anton G.Wu, YoujunKutner, AndrzejWu, Yu-JenKutryb-Zaja̧c, BarbaraWu, ZhihaoKuwahara, TomokiWurm, Jan-PhilipKvasnica, MiroslavWyszkowski, MirosławKwak, BrendaXavier, Nuno M.Kwok, Henry Hang FaiXi, ShuhuaKwon, InchanXi, XinpingKwong, Jennifer Q.Xia, JunKyei, George BXiao, XiangweiLa Rosa, CarmeloXie, BingxianLabilloy, AnataliaXie, MinLaboisse, Christian L.Xie, MouzheLabrador, MaríaXie, TingLabruère, RaphaëlXie, Wei-dongLacadena, JavierXie, WenjunLacatusu, Cristina MihaelaXIRODIMAS, DmitrisLachenmeier, DirkXu, KuiLachowiec, JenniferXu, PengLacina, LukasXu, ShengliLacko, AndrasXu, WenyanLacruz, RodrigoXu, XinLafite, PierreXu, XuehuaLafond, MickaëlXu, YanLafont, ElodieYadav, PramodLafontaine, DenisYadav, RachitaLaforenza, UmbertoYahiroda, HidekiLaganà, Antonio SimoneYakhine-Diop, Sokhna M.SLagares, AlfonsoYamada, HiroshiLagares, DavidYamada, ShuheiLagerstedt, Jens O.Yamamoto, NorioLagos, LeidyYamamoto, ToshiharuLaguna, AriadnaYamamoto, YuzoLagunas, BeatrizYamamura, HisaoLahiri, TanayaYamasaki, TriciaLalas, StavrosYamashita, HitoshiLam, Sio-HongYamashita, SatoshiLambeau, GerardYamauchi, SatoshiLambrev, Petar H.Yan, DayunLamichhane, RajanYan, Y. SandersLampe, PaulYanai, KazuhikoLamy, ElsaYang, BoLancel, SteveYang, Chao-HsunLandi, SilviaYang, Chia-NingLandreau, AnneYang, ChunzhangLandrieu, IsabelleYang, GuangdongLanducci, ElisaYang, JinghuaLanekoff, IngelaYang, KyungaeLangdon, SimonYang, Shun-FaLangella, EmmaYang, YongkangLanza, GiuseppeYang, Zeng-QuanLaradi, SandrineYang, ZiyinLario, Luciana DanielaYano, AkiraLaroche, CélineYao, ChaoqunLaRue, Clayton T.Yao, LiLasonder, EdwinYarema, KevinLasorsa, Francesco MassimoYarlett, NigelLatek, DorotaYasuda, KazukiLattanzi, SimonaYasuhiko, KogaLattanzio, RossanoYasunaga, MasahiroLau-Cam, Cesar A.Yates, JohnLaurini, ErikYazawa, TakashiLauro, DavideYe, Jian-HuiLavra, LucaYe, ShigenLaw, RubyYe, Xing-guoŁawicki, Sławomir ł.Yedjou, Clement G.Lawrence, MichaelYeh, I-JuLazarus, MajaYi, Sun-ShinLe Hir, HervéYi, TaoLe, Nguyen Quoc KhanhYildirim-Ayan, EdaLeadlay, PeterYin, Mei-ChinLeaver, MichaelYlostalo, Joni H.Lebaron, RichardYochum, Gregory S.Leblond, JeffreyYohda, MasafumiLebrun, Marc-HenriYokoi, FumiakiLederkremer, Gerardo Z.Yonekura, ShinichiLee, Bong HoYoon, Joon-KeeLee, Bor-ShiunnYoon, YisangLee, Byoung DaeYoshikawa, KenichiLee, Chang HoonYoshimatsu, TakeshiLee, Chia-HwaYoshimura, MasamiLee, Dae-SungYou, YoungjaeLee, Eun SeongYounger, ScottLee, GabsangYu, Cheng-JuLee, Gun WooYu, DanxiaLee, Hee SeungYu, Deng-GuangLee, I-TaYu, Han-GangLee, JaeminYu, JianLee, JintaeYu, Lu-GangLee, Kuo HaoYu, Tsyr-YanLee, KwanukYu, VictorLee, Man RyulYu, Yu-HsiangLee, Mi KyeongYuan, GailingLee, Min YoungYuan, QingbinLee, Sang GilYuan, XuegangLee, Shao-ChenYuan, ZhihongLee, SukmookYuann, Jeu-Ming P.Lee, Sung-HyenYucesan, GundogLee, Wei-ChiehYudoh, KazuoLee, Yen ChienYuguchi, YoshiakiLee, Yeon SunYukl, Erik T.Lee, YounbokYumoto, HiromichiLee, Yu-HsuanYurchenko, EkaterinaLeem, JaechanYurina, Nadezhda P.Leeper, ThomasYutzey, KatherineLees, Watson J.Zacconi, FlaviaLeffler, HakonZagotto, GiuseppeLegembre, PatrickZaharia, ValentinLegrand, FannyZahoranová, AnnaLehotsky, JanZaichenko, Olexandr S.Lemasters, John J.Zaid, HilalLemli, BeataZakiyanov, OskarLemma, EnricoZakrocka, IzabelaLenaghan, ScottZalewska, AnnaLeng, RogerZalubovskis, RavisLeng, SiyangZam, WissamLeon, FranciscoZambito, YleniaLeonardi, SalvatoreZambrano, AngaraLeondaritis, GeorgeZamfir, MedanaLeppik, LiudmilaZamparini, FaustoLerma, ClaudiaZanotti, GiuseppeLes, FranciscoZapico, Sara C.Lesgards, Jean-FrançoisZappavigna, SilviaLeslie, Andrew G.W.Zappulla, DavidLesnefsky, Edward J.Zarschler, KristofLešnik, SamoŻarski, DanielLesyk, Roman B.Zarza, XavierLetek, MichalZawacka-Pankau, JoannaLeu, SteveZeigler, DanielLeung, Yu minZeković, ZoranLevi, GiovanniŻelaszczyk, DorotaLevick, ScottZelkó, RománaLevy, Galit KatarivasZenarruzabeitia, OlatzLewczuk, BogdanZhang, ErshuaiLewis, Aurélia E.Zhang, FanLewis, Dale E.A.Zhang, FumingLewis, GladiusZhang, JiLeybaert, LucZhang, JianLeychenko, ElenaZhang, JianyingLi, ChenshuangZhang, PengfeiLi, Chien-FengZhang, QuanLi, ChunyuanZhang, WencaiLi, GuohuiZhang, WenxuanLi, HaoboZhang, XiaodongLi, HeZhang, XiaonanLi, Jiao JiaoZhang, YixuanLi, JibinZhang, YonghongLI, JieZhang, ZhaoLi, JunhaoZhang, ZhibingLi, Kuo-BinZhang, ZhiguoLi, LianboZhao, HuixianLi, LinZhao, LingLi, QuanxiZhao, NingLi, ShantaoZhao, NingningLi, WeiZhao, QianLi, XiaogangZhao, YangLi, XueZhao, YuguangLi, YanZhao, YutongLiang, Nu-ChuZheng, WenweiLiang, TingmingZheng, Yun-WenLiang, YanZheng, ZhongLiao, Hsuan-ChiehZhou, BangjunLiao, Jiunn-WangZhou, ChiLiao, PanZhou, HaoLiao, ZehuanZhou, Heshan SamLiaudanskas, MindaugasZhou, HuaLiaw, LucyZhou, JinrongLiberante, Fabio GiuseppeZhou, LiangLiberato, Lorenzo DiZhou, MinLibich, DavidZhou, PhoebeLiby, KarenZhou, QiminLicea, AlexeiZhou, QingyuLicinia, Gouveia ZeliaZhou, TianhaoLiebscher, SandraZhou, YubinLikhatskaya, GalinaZhou, ZhidongLi-Kroeger, DavidZhu, LinLiland, NinaZhu, PengxiangLilly, BrendaZhu, Qiuyu MartinLim, Chinten JamesZhu, QuinnLim, Jae HyangZhu, ShiweiLim, Julie Ching-HsiaZhu, ShujiaLim, KyuZhu, WufuLim, Lee WeiZhukov, IgorLim, YoonghoZhukova, NataliaLimaye, VidyaZhuo, ChunliuLimongelli, GiuseppeZidar, NinaLin, Chih-LiZidek, JanLin, Chi-WenZiemann, MarkLin, FanZille, AndreaLin, HongjunZimmermann, SonjaLin, Hsiang-YuZinta, GauravLin, Hsun-HsunZizza, PasqualeLin, Jeng-ShaneZloh, MireLin, Nien TsungZłotkowska, DagmaraLin, Shih-ChiehZmijewski, MichalLin, Tzer-BinZographos, S. E.Lin, Yi-TingZoidl, GeorgLin, ZhichengZolfaghari Emameh, RezaLinacero, RosarioZondler, LisaLinares-Pastén, JavierZórad, StefanLinciano, PasqualeZou, XueqingLindahl, UlfZovko Končić, MarijanaLinder, BenediktZubair, Abba C.Linder, StigZunk, MatthewLindsey-Boltz, Laura A.Zupan, JanjaLinsenmeier, Robert A.Zuppinger, ChristianLionetti, VincenzoZuverza-Mena, NubiaLiou, Jr-JiunZuza, EsterLipińska, BarbaraZylinska, LudmilaLipscomb, Michael W.


